# Vascular defects associated with hereditary hemorrhagic telangiectasia revealed in patient-derived isogenic iPSCs in 3D vessels on chip

**DOI:** 10.1016/j.stemcr.2022.05.022

**Published:** 2022-06-30

**Authors:** Valeria V. Orlova, Dennis M. Nahon, Amy Cochrane, Xu Cao, Christian Freund, Francijna van den Hil, Cornelius J.J. Westermann, Repke J. Snijder, Johannes Kristian Ploos van Amstel, Peter ten Dijke, Franck Lebrin, Hans-Jurgen Mager, Christine L. Mummery

**Affiliations:** 1 Department of Anatomy and Embryology, Leiden University Medical Center, Leiden 2333ZA, the Netherlands; 2 Department of Anatomy and Embryology and Human iPSC Hotel, Leiden University Medical Center, Leiden 2333ZA, the Netherlands; 3 St. Antonius Hospital, Nieuwegein, the Netherlands; 4 Department of Genetics, University Medical Center, Utrecht, the Netherlands; 5 Department of Cell and Chemical Biology, Oncode Institute, Leiden University Medical Center, Leiden 2333ZA, the Netherlands; 6 Einthoven Laboratory for Experimental Vascular Medicine, Department of Internal Medicine, Leiden University Medical Center, Leiden, the Netherlands; 7 INSERM U1273, ESPCI, CNRS FRE 2031, Paris, France

**Keywords:** hiPSC-derived endothelial cells, hiPSC-ECs, hereditary disorder, blood vessels, hereditary hemorrhagic telangiectasia, HHT1, ENG, microfluidics, vessels on chip, disease modeling

## Abstract

Hereditary hemorrhagic telangiectasia (HHT) is a genetic disease characterized by weak blood vessels. HHT1 is caused by mutations in the *ENDOGLIN* (*ENG*) gene. Here, we generated induced pluripotent stem cells (hiPSCs) from a patient with rare mosaic HHT1 with tissues containing both mutant (*ENG*^c.1678C>T^) and normal cells, enabling derivation of isogenic diseased and healthy hiPSCs, respectively. We showed reduced ENG expression in HHT1 endothelial cells (HHT1-hiPSC-ECs), reflecting haploinsufficiency. HHT1^c.1678C>T^-hiPSC-ECs and the healthy isogenic control behaved similarly in two-dimensional (2D) culture, forming functionally indistinguishable vascular networks. However, when grown in 3D organ-on-chip devices under microfluidic flow, lumenized vessels formed in which defective vascular organization was evident: interaction between inner ECs and surrounding pericytes was decreased, and there was evidence for vascular leakage. Organs on chip thus revealed features of HHT in hiPSC-derived blood vessels that were not evident in conventional 2D assays.

## Highlights

• Vessels from isogenic hiPSCs from HHT1 patients compared
• HHT1-hiPSC-ECs show defective vascular organization in 3D microfluidic chips
• HHT1-hiPSC-ECs show defective EC-pericyte interaction

## Graphical abstract

**Figure:**
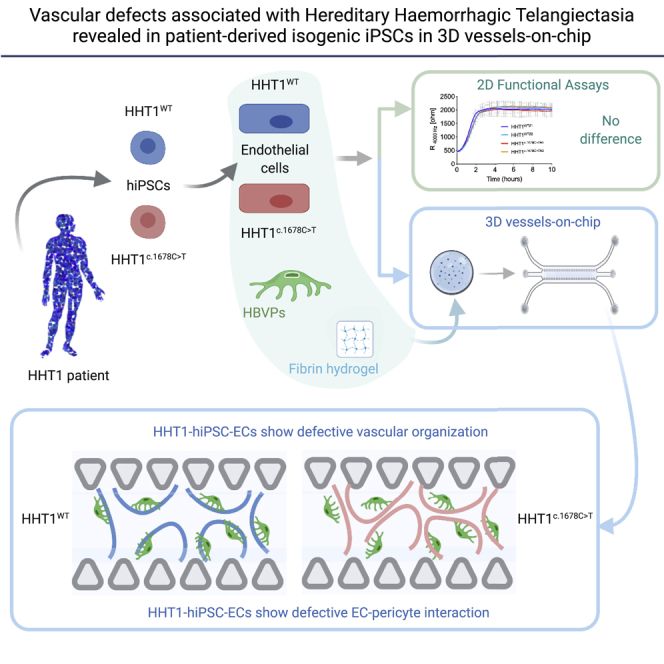

## Teaser

In this article, Orlova and colleagues describe 3D vessels on chip (VoCs) composed of hiPSC-ECs from a patient with hereditary hemorrhagic telangiectasia (HHT1). HHT1-hiPSC-ECs and healthy isogenic controls behaved similarly in 2D culture, but defective vascular organization and reduced pericyte coverage were evident in HHT1-hiPSC-ECs in VoCs. The model is thus a valuable tool for mechanistic studies and future drug discovery.

## Introduction

Hereditary hemorrhagic telangiectasia (HHT) is an inherited genetic disorder caused by autosomal dominant mutations in Endoglin (*ENG*; HHT1), Activin receptor like kinase-1 (*ACVRL1*; HHT2) or *SMAD4* (HHT3), genes that mediate signaling by transforming growth factor β (TGF-β) and bone morphogenetic protein (BMP) in vascular endothelial cells (ECs) (Goumans et al., 2009). Phenotypically, HHT causes tortuous defects in blood vessels, particularly evident in the skin and mucous membranes, that are prone to hemorrhage (Govani and Shovlin, 2009). These abnormalities, called telangiectases, consist of enlarged and dilated capillaries that lack the pericyte/smooth muscle cell coverage of normal vessels. Studies in mice indicated that ENG deficiency can lead to abnormal endothelial-pericyte cell interactions caused by defective paracrine signaling by ECs lacking ENG (Carvalho et al., 2004; Lebrin et al., 2010). More severe abnormalities, evident as arteriovenous malformations (AVMs) in the brain, lung, liver, and gastrointestinal tract, can be fatal if hemorrhage occurs (Govani and Shovlin, 2009). To date, there are no therapies that prevent the formation of these abnormalities in patients with HHT or reverse them once they have occurred. At most, current therapies, such as surgical intervention or cauterization of vessels to divert blood flow, ameliorate symptoms of the disease but are not cures (Shovlin, 2010). Medical treatments under investigation include anti-angiogenic, -inflammatory, and -fungal drugs such as humanized anti-vascular endothelial growth factor (VEGF) antibody (bevacizumab) (Dupuis-Girod et al., 2012), thalidomide (Lebrin et al., 2010), itraconazole (Kroon et al., 2020), and other drugs (reviewed in Snodgrass et al., 2021). Genetic models of HHT in mice, in which the genes responsible for the disease in humans are deleted, show clear vascular defects, but they have not shed much light on the specific genotype/phenotype relationships in HHT patients (Tual-Chalot et al., 2015). Attempts to model HHT using primary human umbilical vein ECs (HUVECs) isolated from newborn HHT patients failed to recapitulate the phenotype (Chan et al., 2004). Blood outgrowth ECs (BOECs) or peripheral blood monocytes (PBMCs) from patients with HHT could be alternative sources of cells to model HHT (Begbie et al., 2003; Fernandez-L et al., 2005; Laake et al., 2006), but their poor proliferation *in vitro* makes them unsuitable as a renewable source of ECs for reproducibly modeling the disease in humans and for drug discovery.

In the present study, we aimed to establish an efficient and scalable system that would recapitulate the formation of defective blood vessels in patients with HHT, based on patient-derived human induced pluripotent stem cells (HHT1-hiPSCs). We hypothesized that HHT1-hiPSCs might be useful for (1) identifying mechanisms underlying disease predisposition and modeling clinical features of HHT1 *in vitro* and (2) investigating defective endothelial-pericyte interactions.

## Results

### HHT1-hiPSC-ECs reflect ENG haploinsufficiency with no apparent differences in functionality

**Figure 1:**
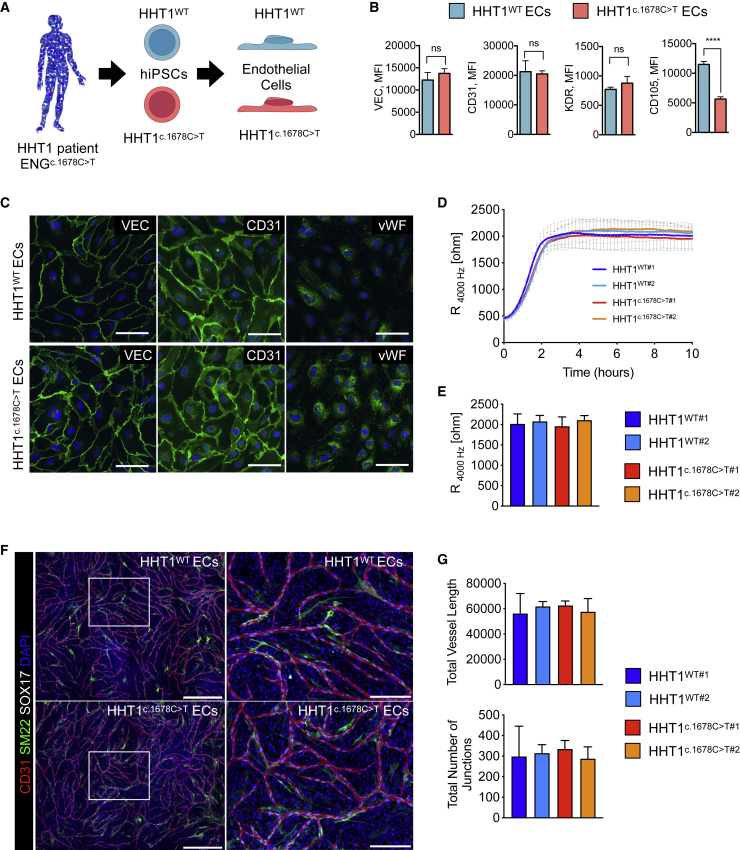
Characterization of HHT1-hiPSC-ECs (A) Schematic overview of ECs differentiated from HHT1 patient-derived isogenic hiPSCs. (B) FACS analysis of surface VEC, CD31, KDR, and ENG (CD105) expression on isolated ECs (P2) from HHT1^WT^ and HHT1^c.1678C>T^ hiPSC lines. ECs differentiated from three independent hiPSC clones were analyzed. Median fluorescent intensity values are shown. Error bars are ± SD. Unpaired t test. ^∗∗∗∗^p < 0.001. (C) Immunofluorescent analysis of EC markers VEC, CD31, and vWF on isolated ECs from HHT1 patient-derived isogenic hiPSCs (P2). Scale bar: 75μm. (D) Absolute resistance of the EC monolayer at 4,000 Hz in complete EC growth medium (CGM). ECs differentiated from two independent hiPSC clones were analyzed. Error bars are ± SD of three to five independent biological experiments per clone. (E) Quantification of absolute resistance values at 4,000 Hz from (D). Error bars are shown as ± SD of five independent biological experiments. (F) Representative immunofluorescent images of an *in vitro* vasculogenesis sprouting assay at day 10 of the co-culture of ECs differentiated from two independent clones of HHT1 patient-derived isogenic hiPSCs and CD31^-^ cells differentiated from an independent control hiPSC line. ECs are stained with anti-CD31 (red) and anti-SOX17 (gray), contractile CD31^-^ cells with anti-SM22 (green), and nuclei with DAPI (blue). Left panels: automatically stitched images (10× objective, 4 × 4 focus planes) are shown; scale bar: 750 μm. Right panels: magnification of the framed area in the left panel. Scale bar: 250 μm. (G) Quantification of EC sprouting network at day 10 of the co-culture. The total vessel length and total number of junctions are shown. Data are shown as ± SD of three independent biological experiments.

hiPSC lines were generated from somatic tissue from a patient with HHT1 with a heterozygous nonsense mutation in *ENG* (NM_001114753.2 (*ENG*):c.1678C>T; p.(Gln560^∗^)), which causes ENG haploinsufficiency (Letteboer et al., 2005). The patient was identified as being a genetic mosaic, allowing generation of isogenic pairs of hiPSC lines with and without the mutation (HHT1^c.1678C>T^ and HHT1^WT^) (Figures S1A–S1C; unpublished data). HHT1-hiPSC clones had normal karyotypes and were verified as pluripotent using standard methods (PluriTest, expression of pluripotency markers and spontaneous differentiation toward three germ cell lineages) (Figures S1D–S1G). HHT1-hiPSCs were then induced to differentiate to ECs (Orlova et al., 2014a; 2014b). Surface ENG (CD105) was significantly downregulated in HHT1^c.1678C>T^-hiPSC-ECs compared with HHT1^WT^-hiPSC-ECs (Figures 1A and 1B). By contrast, surface expression of other major EC markers such as vascular endothelial cadherin (VEC), platelet and endothelial adhesion molecule (CD31/PECAM1), and kinase insert domain receptor (KDR), also known as VEGFR2, was similar among lines (Figure 1B). ENG haploinsufficiency had no apparent effect on the proliferation of HHT1-hiPSC-ECs (Figure S2A). HHT1-hiPSC-ECs showed similar responses upon short-term stimulation with BMP9 and TGF-β, except that *ID1* expression was significantly upregulated in HHT1^c.1678C>T^-hiPSC-ECs after 2 h of TGF-β treatment (Figure S2B).

Immunostaining showed comparable expression and localization of VEC, CD31, and intracellular von Willebrand factor (vWF) (Figure 1C). Barrier function was assessed by real-time impedance spectroscopy (electric cell-substrate impedance sensing [ECIS]) in an integrated assay of electric wound healing for migration, as previously described (Halaidych et al., 2018). Barrier function in two dimension (2D) was similar in healthy and HHT1-hiPSC-ECs in “complete” EC growth medium (CGM) (Figures 1D and 1E), and migration rates were identical (data not shown). Barrier function was increased in growth-factor-free medium (1% platelet-poor plasma serum [PPS]) (Figures S2C and S2D), in line with our previous findings (Halaidych et al., 2018). BMP9 addition reduced barrier function equally in healthy and HHT1^c.1678C>T^-hiPSC-ECs (Figures S2C and S2D). TGF-β addition significantly reduced barrier function in HHT1^c.1678C>T^-hiPSC-ECs but had no significant effect in HHT1^WT^-hiPSC-ECs (Figures S2C and S2D), which is also in line with the differences in responses in HHT1-hiPSC-ECs upon TGF-β treatment. Migration rates were similar in all conditions examined (Figure S2E).

Finally, the ability to form 2D vascular networks *in vitro* was examined, as described previously (Halaidych et al., 2018; Orlova et al., 2014a, 2014b). HHT1-hiPSC-ECs formed well-organized vascular networks when cultured with stromal cells, and these were indistinguishable from healthy controls. Furthermore, stromal cells adjacent to ECs upregulated expression of the contractile smooth muscle cell marker SM22 (Figure 1F). Quantification of vascular networks formed by HHT1^c.1678C>T^-hiPSC-ECs and HHT1^WT^-hiPSC-ECs showed similar total vessel length and number of branches (Figure 1G) as well as total number of SOX17^+^ ECs and number of adjacent SM22^+^ cells (data not shown).

### HHT1-hiPSC-ECs show defective vascular organization in 3D microfluidic chips

**Figure 2:**
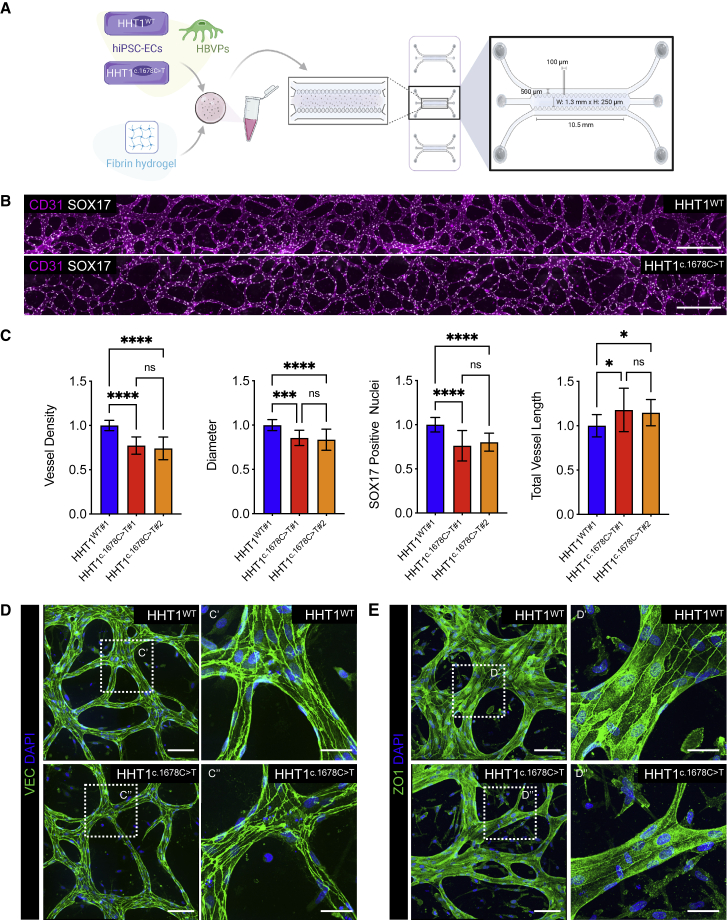
HHT1-hiPSC-ECs show defective vascular organization in 3D microfluidic chips (A) Schematic showing generation of 3D vascular networks in microfluidic chips using hiPSC-ECs and HBVPs. (B) Representative images of vascular networks formed by HHT1-hiPSC-ECs differentiated from HHT1^WT^ and HHT1^c.1678C>T^ hiPSC lines in microfluidic chips. ECs are stained with anti-CD31 (magenta) and anti-SOX17 (yellow). Scale bar: 500 μm. (C) Quantification of HHT1-hiPSC-EC vascular network showing vessel density, diameter, number of HHT1-hiPSC-ECs (SOX17+ nuclei), and total vessel length. Data are shown as ± SD, Unpaired t test. ^∗∗∗∗^p < 0.0001, ^∗∗∗^p < 0.0005, ^∗^p < 0.05, ns, not significant. Normalized values from independent experiments are shown. From N = 3, n = 9; three independent experiments with three microfluidic channels per experiment (HHT^WT#1^, HHT^c.1678C>T#1^). From N = 5, n = 15; five independent experiments with three microfluidic channels per experiment (HHT^WT#1^, HHT^c.1678C>T#2^). (D and E) Representative confocal images showing VEC (D) and ZO1 (E). Nuclei are stained with DAPI (blue). Inserts are magnifications of framed areas to show VEC and ZO1 localization. Scale bars: 100 μm (left panels) and 40 μm (right panels).

The ability of HHT1-hiPSC-ECs to form microvascular networks in a 3D vessel-on-chip (VoC) model was then examined (Figure 2A). Primary human brain vascular pericytes (HBVPs) were used to support microvascular-network formation, as described previously (Vila Cuenca et al., 2021). Vascular networks developed around day 2–3 of culture; lumenized microvessels were observed around day 5 (Figure S3A). Microfluidic chips were immunostained with antibodies against CD31/PECAM1 and an EC-specific transcription factor SOX17. The ability to form microvascular networks in microfluidic chips was compromised in HHT1^c.1678C>T^-hiPSC-ECs compared with HHT1^WT^-hiPSC-ECs (Figures 2B and 2C), despite similar initial seeding densities (Figure S3A). Quantification of microvascular networks showed reduced vascular density, diameter of the vessels, and number of ECs (SOX17+ nuclei) and an increase of the total length of the vessels of the networks formed by HHT1^c.1678C>T^-hiPSC-ECs (Figures 2C, S3C and S3D). HHT1^WT^-hiPSC-ECs from two independent hiPSC clones behaved similarly (Figure S3B). Furthermore, proliferation of HHT1^c.1678C>T^-hiPSC-ECs was lower than HHT1^WT^-hiPSC-ECs, as evidenced by fewer EdU-positive nuclei (Figures S3E and S3F). To demonstrate that the microvascular networks formed by HHT1-hiPSC-ECs were hollow, fluorescent beads were perfused through the vessels (Video S1). Notably, the beads moved at a considerably lower rate in the microvascular networks formed by HHT1^c.1678C>T^-hiPSC-ECs compared with HHT1^WT^-hiPSC-ECs, indicating reduced flow through 3D vessels formed by HHT1^c.1678C>T^-hiPSC-ECs.

Video S1. Perfusion of fluorescent beads in 3D vessels formed by HHT1WT-hiPSC-ECs and HHT1c.1678C>T-hiPSC-ECs, related to Figure 2

Junctional integrity was examined by immunostaining of the microvascular networks with VEC and ZO1 (Figures 2D and 2E). The results showed that although VEC junctional distribution was comparable, junctional distribution of ZO1 was reduced in the microvascular networks formed by HHT1^c.1678C>T^-hiPSC-ECs compared with HHT1^WT^-hiPSC-ECs.

### HHT1-hiPSC-ECs show defective EC-pericyte interaction

**Figure 3:**
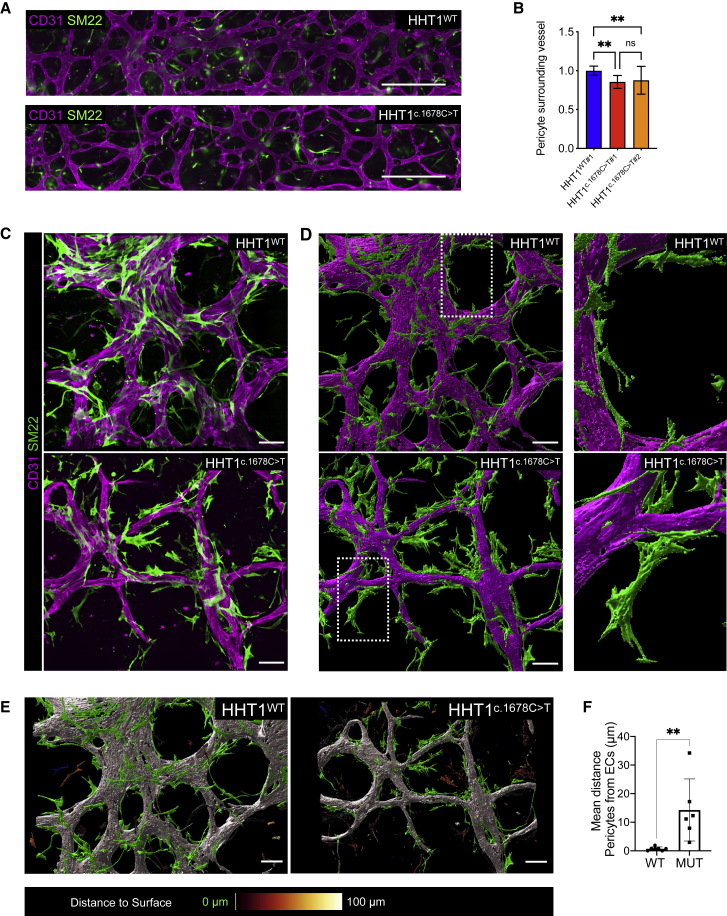
HHT1-hiPSC-ECs show defective EC-pericyte cell interaction (A) Representative images of vascular networks formed by HHT1-hiPSC-ECs differentiated from HHT1^WT^ and HHT1^c.1678C>T^ hiPSC lines in microfluidic chips. ECs are stained with anti-CD31 (magenta) and anti-SM22 (green). Scale bar: 500 μm. (B) Quantification of percentage of pericytes surrounding vessel and average length of pericytes using CellProfiler. Normalized values from independent experiments are shown. From N = 3, n = 9; three independent experiments with three microfluidic channels per experiment (HHT^WT#1^, HHT^c.1678C>T#1^). From N = 5, n = 15; five independent experiments with three microfluidic channels per experiment (HHT^WT#1^, HHT^c.1678C>T#2^). Error bars are ± SD. Unpaired t test. ^∗∗^p < 0.005. (C) Representative spinning disk confocal images of vascular networks of HHT1-hiPSC-ECs differentiated from HHT1^WT^ and HHT1^c.1678C>T^ hiPSC lines and HBVPs in 3D microfluidic chips with hiPSC-EC (magenta; CD31) and HBVPs (green; SM22). Scale bar: 100 μm. (D) Left panels: surface rendering of images in (B) processed using IMARIS 9.5 software (Bitplane, Oxford Instruments). Scale bar: 100 μm. Right panels: magnification of the framed area in the left panel. (E) Surface-rendering images processed using Imaris 9.5 software (Bitplane, Oxford Instruments) from spinning disk confocal images showing vascular networks formed by HHT1-hiPSC-ECs (gray; CD31) differentiated from HHT1^WT^ and HHT1^c.1678C>T^ hiPSC lines in microfluidic chips. Color code for HBVPs showing green for objects touching the vessel, and color scale representing distance from the vessel. Scale bar: 30 μm. (F) Quantification of average distance of surface-rendered SM22 cells to CD31 surface-rendered objects using IMARIS 9.5 software (Bitplane, Oxford Instruments). From N = 5, n = 7 (wild type [WT]) and n = 6 (mutant [MUT]); five independent experiments with one to three areas of each channel quantified. Error bars are ± SD. Unpaired t test. ^∗∗^p < 0.01.

We next analyzed the interaction between HHT1-hiPSC-ECs and HBVPs in the VoC model. Microfluidic chips were immunostained with antibodies against CD31/PECAM1 and the contractile smooth muscle cell marker SM22 (Figure 3A). Quantification of microvascular networks formed by HHT1^c.1678C>T^-hiPSC-ECs showed reduced pericyte coverage compared with HHT1^WT^-hiPSC-ECs (Figures 3B, S4A, and S4B). HHT1^c.1678C>T^-hiPSC-ECs also showed increased pericyte distance from ECs when compared with HHT1^WT^-hiPSC-ECs (Figures 3C and 3D). Surface rendering of confocal images revealed that pericytes in vascular segments formed by HHT1^c.1678C>T^-hiPSC-ECs were positioned more distally from ECs compared with vascular segments formed by HHT1^WT^-hiPSC-ECs (Figures 3E and 3F). No significant differences were found in extracellular matrix (ECM) organization in microvascular networks formed by HHT1^WT^-hiPSC-ECs and HHT1^c.1678C>T^-hiPSC-ECs, as demonstrated by counterstaining with VEC and collagen IV (COLIV) (Figures S4C–S4E).

**Figure 4:**
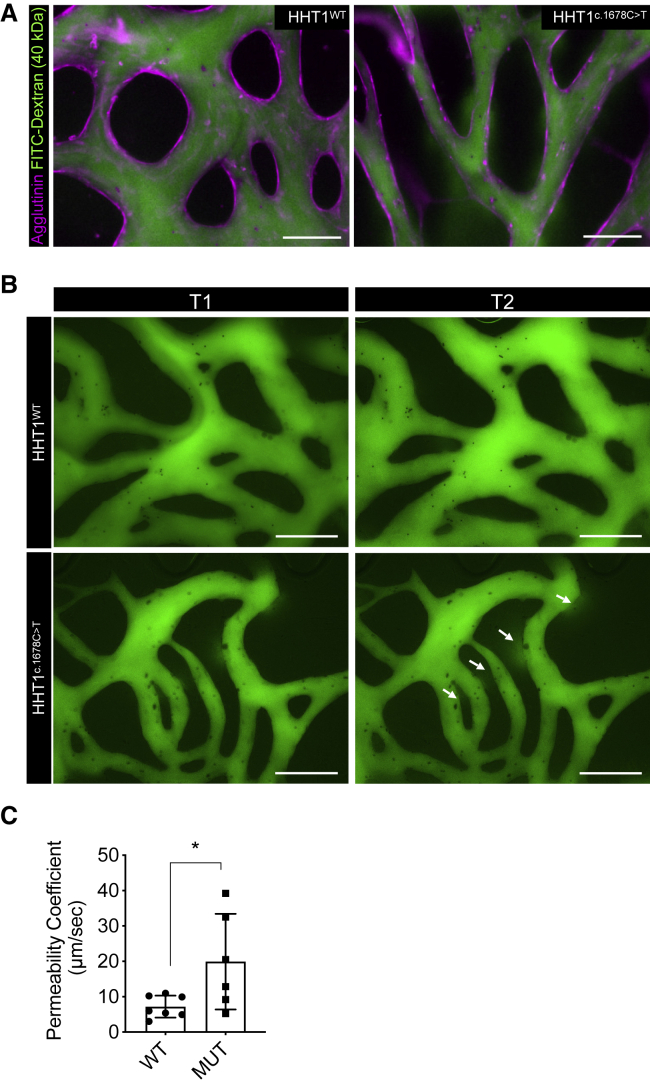
HHT1-hiPSC-ECs form leaky vessels (A) Representative immunofluorescent images of vascular network formed by HHT1-hiPSC-ECs differentiated from HHT1^WT^ and HHT1^c.1678C>T^ hiPSC lines and HBVPs in 3D microfluidic chips with hiPSC-EC (magenta; agglutinin) and dextran (40 kDa, green). 10x (EVOS) scale bar: 200 μm. (B) Representative immunofluorescent images of vascular networks perfused with dextran (40 kDa, green) at two different timepoints (T1 = 0 s and T2 = 30 s). White arrows show dextran leakage in vascular network formed by HHT1^c.1678C>T^ hiPSC-ECs. (C) Quantification of permeability coefficient from N = 3, n = 7; three independent biological replicates with duplicate or triplicate microfluidic channels per experiment. Data are shown as ± SD. Unpaired t test. ^∗^p < 0.05.

We next performed a fluorescent dextran leakage assay to test whether reduced EC-pericyte interaction caused the 3D vessels to be more fragile and prone to leak. Fluorescently labeled dextran (FITC-Dextran, 40 kDa) was added into the medium channel of the organ-on-chip device, and real-time videos of vascular segments pre-stained using fluorescent agglutinin were made (Figure 4A; Video S2). Quantification of permeability coefficient showed increased leakage of 3D vascular segments formed from HHT1^c.1678C>T^-hiPSC-ECs compared with HHT1^WT^-hiPSC-ECs (Figures 4B and 4C).

Video S2. Perfusion of FITC-Dextran (40 kDa) in 3D vessels formed by HHT1WT-hiPSC-ECs and HHT1c.1678C>T-hiPSC-ECs, related to Figure 4

## Discussion

In this study, we developed a human *in vitro* model for the genetic vascular disorder HHT using hiPSCs derived from patients with mutations in the *ENG* gene (HHT1). The results showed that we likely captured the direct effects of reduced ENG protein on the EC surface without compensation or adaption mechanisms that normally occur *in vivo,* notably in mutant mice (El-Brolosy and Stainier, 2017). Thus, even though differences in vessels were observed, aspects of the HHT phenotype were masked although the poor EC-pericyte interaction was similar to that reported previously in Eng^+/-^ mutant mice (Galaris et al., 2019). This would contribute to vessel instability and could cause the leaky 3D vascular network formed by HHT1^c.1678C>T^-hiPSC-ECs, in line with observations in patients.

The results in the HHT1-hiPSC-EC 2D model differ from earlier studies in which small interfering RNA (siRNA) was used to knock down *Eng* transiently in mouse embryonic ECs (Lebrin et al., 2004)*.* Complete *Eng* knockdown in mouse embryonic ECs resulted in reduced EC proliferation and TGF-β signaling in 2D assays. On the other hand, *ENG* haploinsufficiency had no effect on EC function in 2D, with proliferation, barrier function, and sprouting angiogenesis in ECs derived from HHT1^c.1678C>T^-hiPSC clones indistinguishable from isogenic controls.

To establish a HHT1-hiPSC-ECs VoC model, we used a commercially available microfluidic chip that supports formation of an interconnected microvascular network (Campisi et al., 2018; Chen et al., 2017; Shin et al., 2012, Vila Cuenca et al., 2021). The model allows simultaneous analysis of both the early steps of the 3D vascular-network formation, such as EC cell proliferation, migration, lumen formation, remodeling and pruning (regression), and endpoint analysis. This includes high-resolution microscopy for EC-pericyte interaction, perfusion studies, and vascular leakage assays. Gravity-driven flow in these chips is sufficient for the maintenance of the vascular segments that are perfused with non-perfused vascular segments regressing overtime, similar to what was observed *in vivo* (Franco et al., 2015; Kochhan et al., 2013).

Overall, we found that HHT1^c.1678C>T^-hiPSC-ECs showed multiple similarities to Eng^+/-^ mutant mice (Arthur et al., 2000; Carvalho et al., 2004; Lebrin et al., 2010), although there were some differences. These included the formation of narrower vessels with fewer ECs by HHT1^c.1678C>T^-hiPSC-ECs than healthy controls. Furthermore, HHT^c.1678C>T^-hiPSC-ECs showed reduced junctional localization of ZO1, although localization of VEC was comparable. This could be a result of reduced EC-matrix adhesion, which in turn could affect cell-cell junctions (Yamamoto et al., 2015) and reduced perfusion and increased regression of vascular networks, as described previously in *eng* mutant zebrafish (Sugden et al., 2017), resulting in reduced EC-pericyte interaction. However, the role of ENG in regulation of cell-to-cell and cell-to-matrix adhesion is beyond the scope of the present study. Thus, despite some shortcomings of the VoC model in capturing the complete HHT patient phenotype, we believe the model is a valuable tool to investigate underlying causes of poor EC-pericyte interaction and identify drugs to reverse it and mediate vascular normalization.

Additional triggers of AVM formation include somatic mutations that reduce ENG function, local loss of ENG protein caused by inflammation, and pro-angiogenic triggers (Mahmoud et al., 2010; Tual-Chalot et al., 2015). Loss of ENG function in mutant mice was shown to induce defective migration against blood flow and EC enlargement, which caused vessels to dilate (Sugden et al., 2017). This, in turn, results in higher hemodynamic forces and peripheral hypoxia that support the enlargement of AVMs (Sugden and Siekmann, 2018). Our current model mainly addressed ENG haploinsufficiency due to *ENG* gene defects and lacked the additional triggers that cause ENG loss of function, such as exposure to pro-angiogenic stimuli and hemodynamic force. The particular advantage of using HHT patient-derived hiPSC lines is that they can be engineered to allow inducible *ENG* knock down or degron-based ENG deletion. We expect that this, in combination with incorporation of pro-inflammatory triggers, such as pro-inflammatory macrophages, into the model will allow complete recapitulation of the phenotype in the future, such that these next-generation models can be implemented in screening for new therapeutic interventions and drug discovery using mechanism-based approaches with opportunities for validation using ECs from patient-derived hiPSCs.

## Experimental procedures

### HHT1 patient-derived hiPSC lines

Biopsies were taken with an informed consent at the St. Antonius Hospital (Nieuwegein, the Netherlands). The generation of the lines was approved by the Leiden University ethics committee under the P13.080 “Parapluprotocol: hiPSC.” Patient samples, fibroblasts from skin biopsies, and erythroblasts isolated from peripheral blood were used for reprogramming. Reprogramming with episomal vectors was done as described, except that a newer generation of vectors without *TP53* shRNA were used (Okita et al., 2011). hiPSCs were routinely cultured on Matrigel (BD) in mTeSR1 and/or on Vitronectin XF in TeSR-E8 (all from Stem Cell Technologies) according to the manufacturer’s protocol. Standard characterization of hiPSCs was performed as described previously (Bouma et al., 2017, 2020; Dambrot et al., 2013; Freund et al., 2010). Karyotype analysis of undifferentiated cells was performed using combined binary ratio labeling-fluorescence *in situ* hybridization (COBRA-FISH) (Szuhai and Tanke, 2006), and pluripotency of the hiPSC clones was confirmed by PluriTest, immunofluorescent staining for OCT3/4, SSEA-4, NANOG, and TRA-1-81, and spontaneous differentiation toward three germ lineages. Sample identity has been confirmed by analysis with the DNA analysis software GeneMarker v.2.6.0 (SoftGenetics, State College, PA, USA) of fragments generated by the AmpFlSTR Profiler Plus PCR Amplification Kit (Applied Biosystems, Foster City, CA, USA) that have been run on a 3730 DNA Analyzer (Applied Biosystems). All tests were performed according to the instructions of the manufacturers.

### Statistics

One-way ANOVA and non-parametric Student’s t test for unpaired measurements were applied as appropriate to test for differences in means between the groups. Detailed statistics are indicated in each figure legend. Data are expressed and plotted as the mean ± SD. Statistical significance is indicated in each figure legend. Statistical analysis was performed with GraphPad Prism 9.0.2.

## Author contributions

V.V.O., designed the research, established functional assays, performed experiments, and wrote the manuscript; D.M.N. and A.C., performed experiments in 3D vascular chips, did imaging, and performed quantification; X.C., performed EC differentiation; C.F., performed reprogramming experiments; F.v.d.H., conducted EC differentiation and isolation and FACS; F.L., assisted with quantification of microfluidic experiments; C.J.J.W., R.J.S., and H.-J.M., provided HHT patient samples; J.K.P.v.A., conducted genetic analysis; P.t.D. and F.L. helped analyze the data; C.L.M., designed the research and wrote the manuscript.

## Declaration of interests

The authors declare no competing interests.

## Supplemental information

Document S1. Figures S1–S4, Tables S1 and S2, and Supplemental experimental procedures

Document S2. Article plus supplemental information
