# Supplementary material for: Vascular defects associated with hereditary hemorrhagic telangiectasia revealed in patient-derived isogenic iPSCs in 3D vessels on chip
Source: Stem Cell Reports. 2022 Jun 30;17(7):1536–45. doi: 10.1016/j.stemcr.2022.05.022 (PMC9287680; doi:10.1016/j.stemcr.2022.05.022)
Supplement: Document S1. Figures S1–S4, Tables S1 and S2, and Supplemental experimental procedures [file mmc1.pdf]

Stem Cell Reports, Volume *17*

Supplemental Information

Vascular defects associated with hereditary hemorrhagic telangiecta-sia revealed in patient-derived isogenic iPSCs in 3D vessels on chip

Valeria V. Orlova, Dennis M. Nahon, Amy Cochrane, Xu Cao, Christian Freund, Francijna van den Hil, Cornelius J.J. Westermann, Repke J. Snijder, Johannes Kristian Ploos van Amstel, Peter ten Dijke, Franck Lebrin, Hans-Jurgen Mager, and Christine L. Mummery

Inventory of Supplemental Information

Supplemental Figures and Legends:

Figure S1. Related to Figure 1. Characterization of HHT1 patient-derived hiPSCs.
Figure S2. Related to Figure 1. Characterization of HHT1-hiPSC-ECs.
Figure S3. Related to Figure 2. HHT1-hiPSC-ECs show defective vascular organization in VoC.
Figure S4. Related to Figure 3. HHT1-hiPSC-ECs show defective EC-pericyte cell interaction in VoC.

Supplemental Tables:

Supplemental Table 1. List of FACS antibodies.
Supplemental Table 2. List of antibodies for IF.

Supplemental Videos:

Supplemental Video 1. Perfusion of fluorescent beads in 3D vessels formed by HHT1^{WT}-hiPSC-ECs and HHT1$^{c.1678C>T}$-hiPSC-ECs. Related to Figure 2.

Supplemental Video 2. Perfusion of FITC-Dextran (40 kDa) in 3D vessels formed by HHT1^{WT}-hiPSC-ECs and HHT1$^{c.1678C>T}$-hiPSC-ECs. Related to Figure 4.

Supplemental Experimental Procedures

Supplemental References

SUPPLEMENTAL FIGURE 1.

A

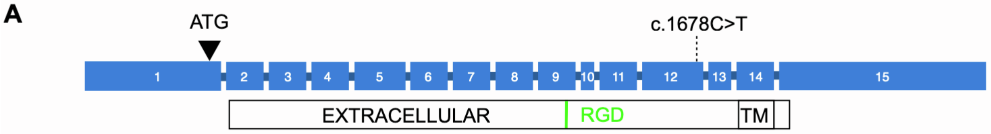

B

hiPSCs	Reprogramming method	Number of clones	Somatic cells	ENG CDS	ENG Protein	Sex	DNA Fingerprinting	LUMC iPSC line
HHT1^{WT}	Episomal	3	Fibroblast	WT	WT	Male	Identical DNA profile: HHT1^{WT} HHT1$^{c.1678C>T}$	LUMC0083iENG
HHT1$^{c.1678C>T}$	Episomal	3	Erythroblast (PBMCs)	NM_001114753.2: c.1678C>T	p.(Gln560*)	Male		LUMC0082iENG

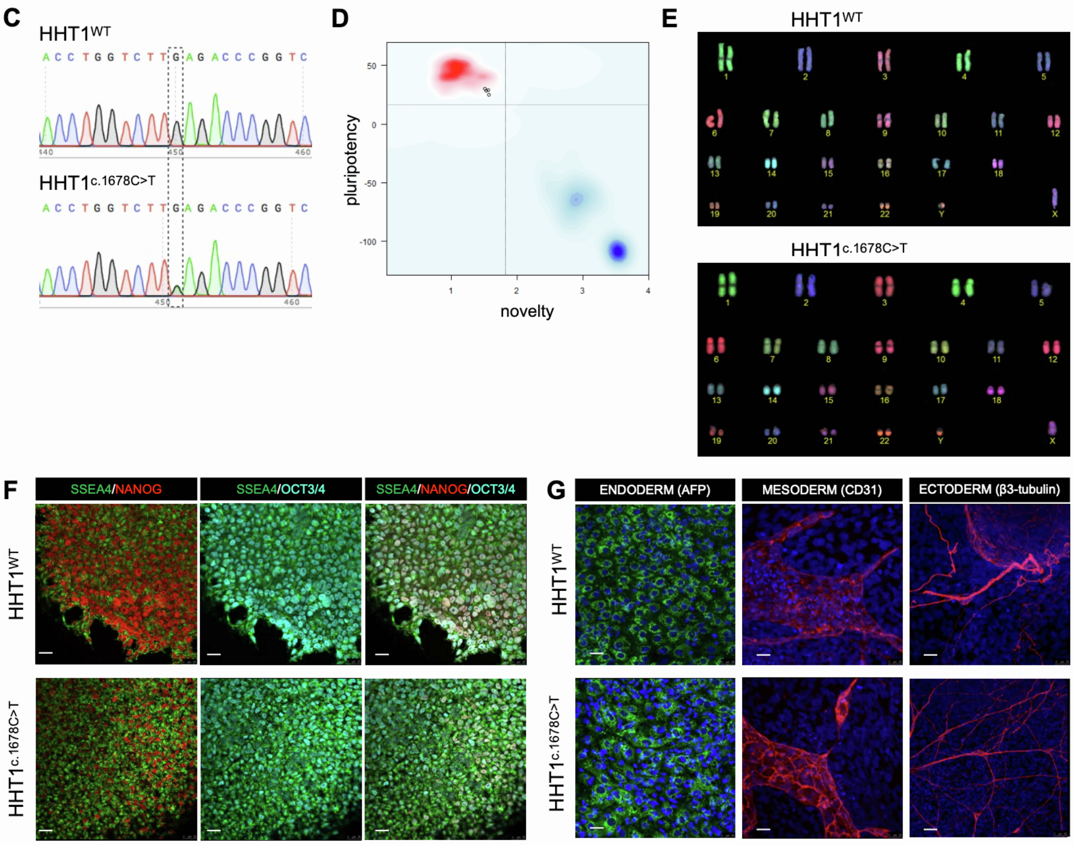

Figure S1. Related to Figure 1. Characterization of HHT1 patient-derived hiPSCs. (A) Schematic overview of *ENG* genomic map, protein map and location of the mutation. (B) Overview of HHT-hiPSC lines. (C) Sanger sequencing of the genomic DNA from HHT1 patient-derived hiPSC lines to confirm the mutation. (D) Bioinformatic assessment of pluripotency of HHT1 patient-derived hiPSCs (PluriTest): two clones per hiPSC line (HHT1^{WT} and HHT1$^{c.1678C>T}$ hiPSCs) were analyzed. (E) COBRA-FISH analysis of karyotype of HHT1 patient-derived hiPSCs. (F) Immunofluorescent images of expression of pluripotency markers: OCT3/4, SSEA4 and Nanog. Scale bar 25 μm. (G) Immunofluorescent images of the spontaneous differentiation demonstrating derivatives of all three germ layers: βIII-tubulin for neuroectoderm, CD31 for mesoderm, AFP for endoderm. Scale bar 25μm.

SUPPLEMENTAL FIGURE 2.

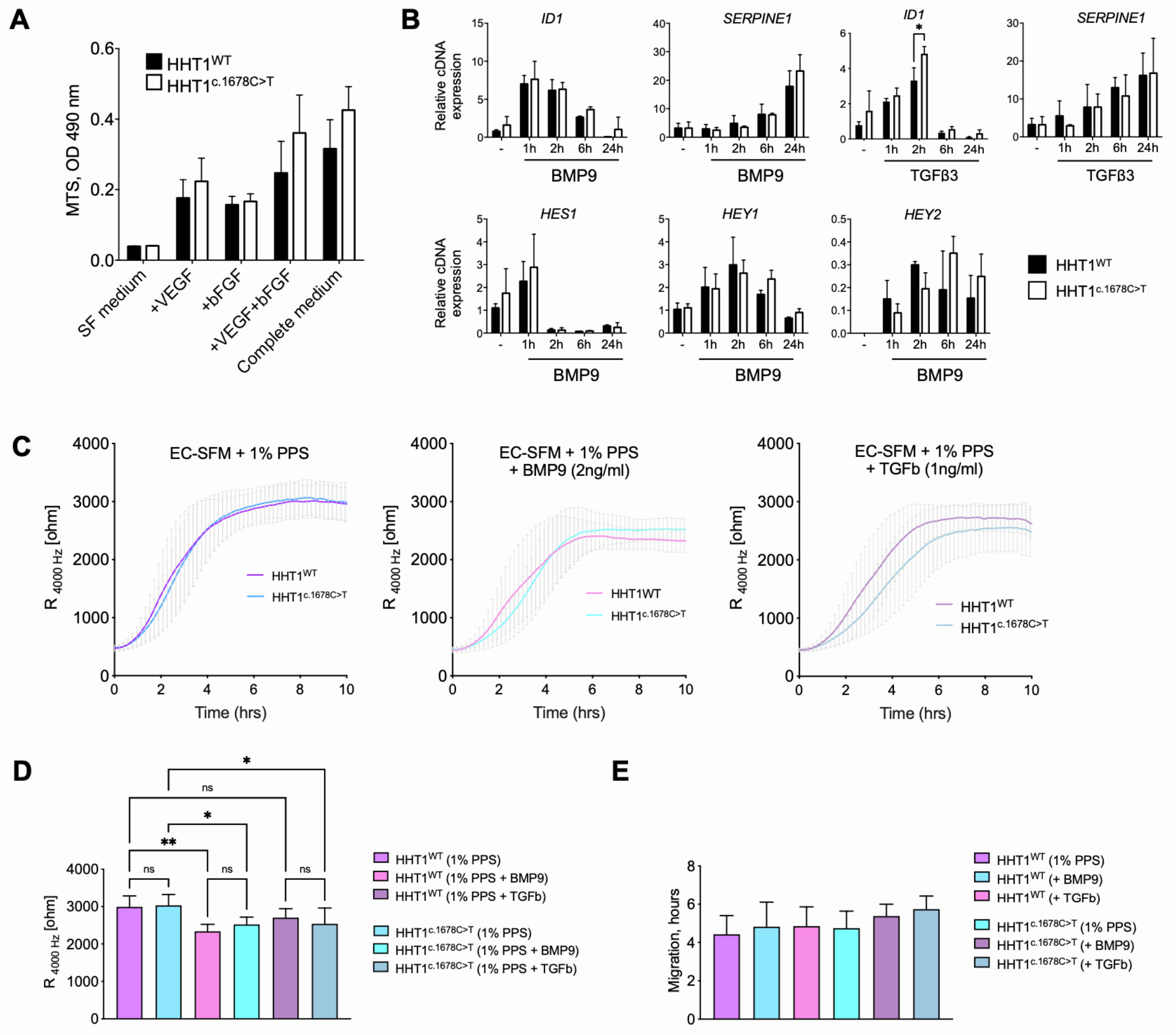

Figure S2. Related to Figure 1. Characterization of HHT1-hiPSC-ECs. (A) Assessment of EC proliferation in serum-free medium (SF medium), SF medium + VEGF (+VEGF), SF medium + bFGF (+FGF), SF medium VEGF + bFGF (+VEGF+FGF) or complete EC growth medium (Complete medium) at 72h after medium change. The analysis was performed on ECs differentiated from three independent clones of HHT1 patient-derived isogenic hiPSCs. Error bars are ±SD. (B) Gene expression analysis of expression of *ID1, SERPINE1, HES1, HEY1 and HEY2* (upon BMP9 1ng/ml stimulation) and *ID1, SERPINE1* (upon TGFβ 1ng/ml stimulation). ECs differentiated from two independent hiPSC clones were analyzed. Data are shown ±SD from three independent experiments. One-way ANOVA. *p<0.05 (C) Absolute resistance of the EC monolayer at 4000 Hz in growth factor-free medium supplemented with 1% PPS (1% PPS), or 1% PPS supplemented with BMP9 (2 ng/ml)(+ BMP9) or 1% PPS supplemented with TGFβ (1 ng/ml)(+ TGFβ) is shown. ECs differentiated from two independent hiPSC clones were analysed. Error bars are ±SD of six independent biological experiments. (D) Quantification of absolute resistance values at 4000 Hz from C. Error bars are shown as ±SD of five independent biological experiments. (E) Quantification of migration rates. Error bars are shown as ±SD of six independent biological experiments. One-way ANOVA. **p<0.01, *p<0.05.

SUPPLEMENTAL FIGURE 3.

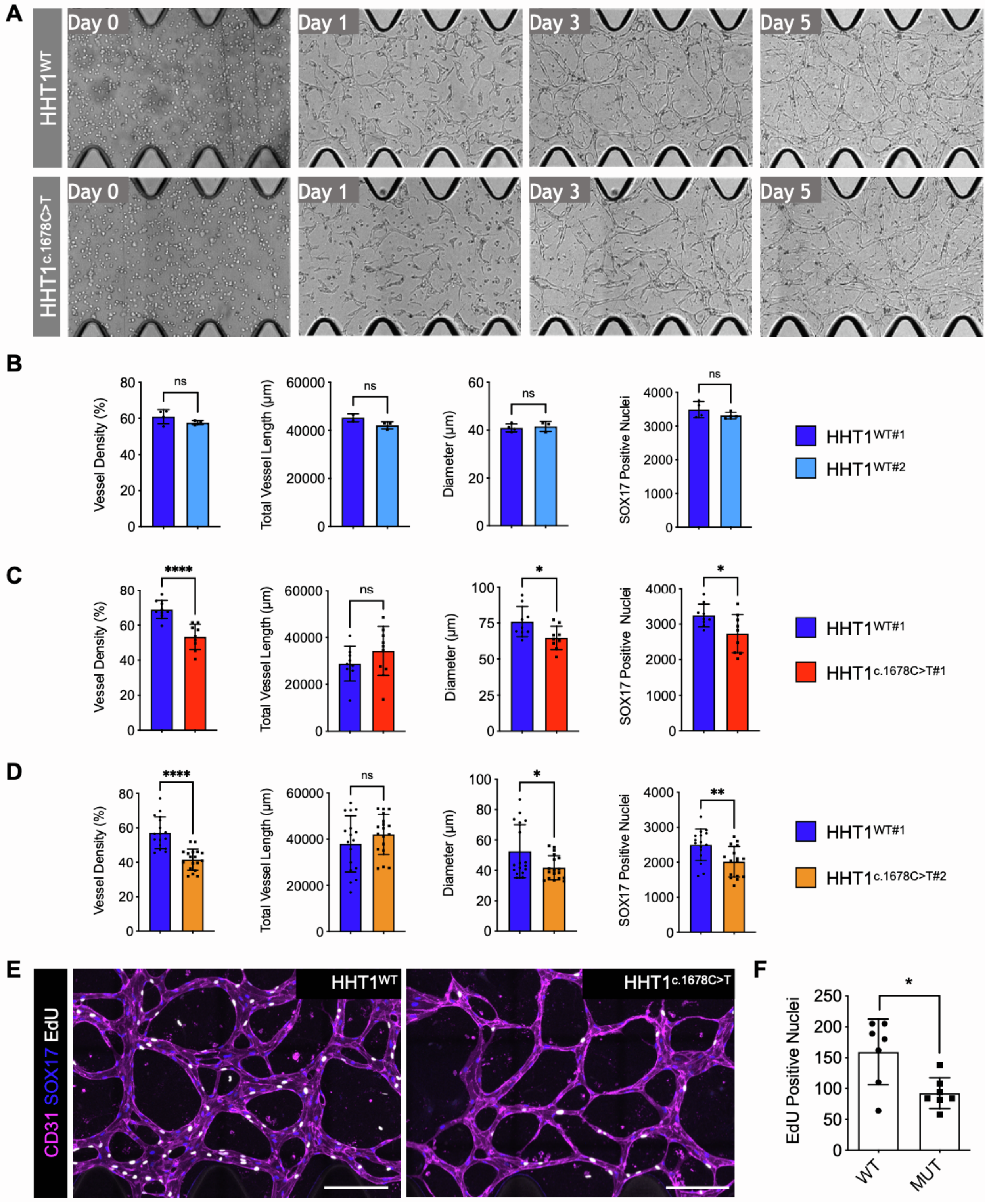

Figure S3. Related to Figure 2. HHT1-hiPSC-ECs show defective vascular organization in VoC. (A) Representative images of microfluidic chips seeded with ECs from HHT1 patient-derived isogenic hiPSCs. Images were taken every 24h from day 0 (after seeding) till day 5. (B) Quantification of HHT1-hiPSC-EC vascular network showing vessel density (%), total vessel length (µm), diameter (µm) and number of HHT1-hiPSC-ECs (SOX17+ nuclei). Data are shown as ±SD, Unpaired t-test. ns = not significant. From N=1, n=3; one independent biological experiment with three microfluidic channels. (C,D) Quantification of HHT1-hiPSC-EC vascular network showing vessel density (%), total vessel length (µm), diameter (µm) and number of HHT1-hiPSC-ECs (SOX17+ nuclei). Data are shown as ±SD, Unpaired t-test. **** p<0.001, *p < 0.05, ns = not significant. From N=3, n=9; three independent biological experimnts with three microfluidic channels per experiment (C). From N=5, n=15; five independent biological experiments (with three microfluidic channels per experiment (D). (E) Representative images showing proliferative (EdU positive) (white) HHT1-hiPSC-ECs differentiated from $HHT1^{WT}$ and $HHT1^{c.1678C>T}$ hiPSC lines and HBVPs in 3D microfluidic chips. Scale bar represents 200 µm. (F) Quantification of proliferative (EdU positive) HHT1-hiPSC-ECs in 3D vascular network in the microfluidic chip. Error bars are ±SD of four microfluidic cultures (one independent biological experiment). Unpaired t-test. * p<0.05.

SUPPLEMENTAL FIGURE 4.

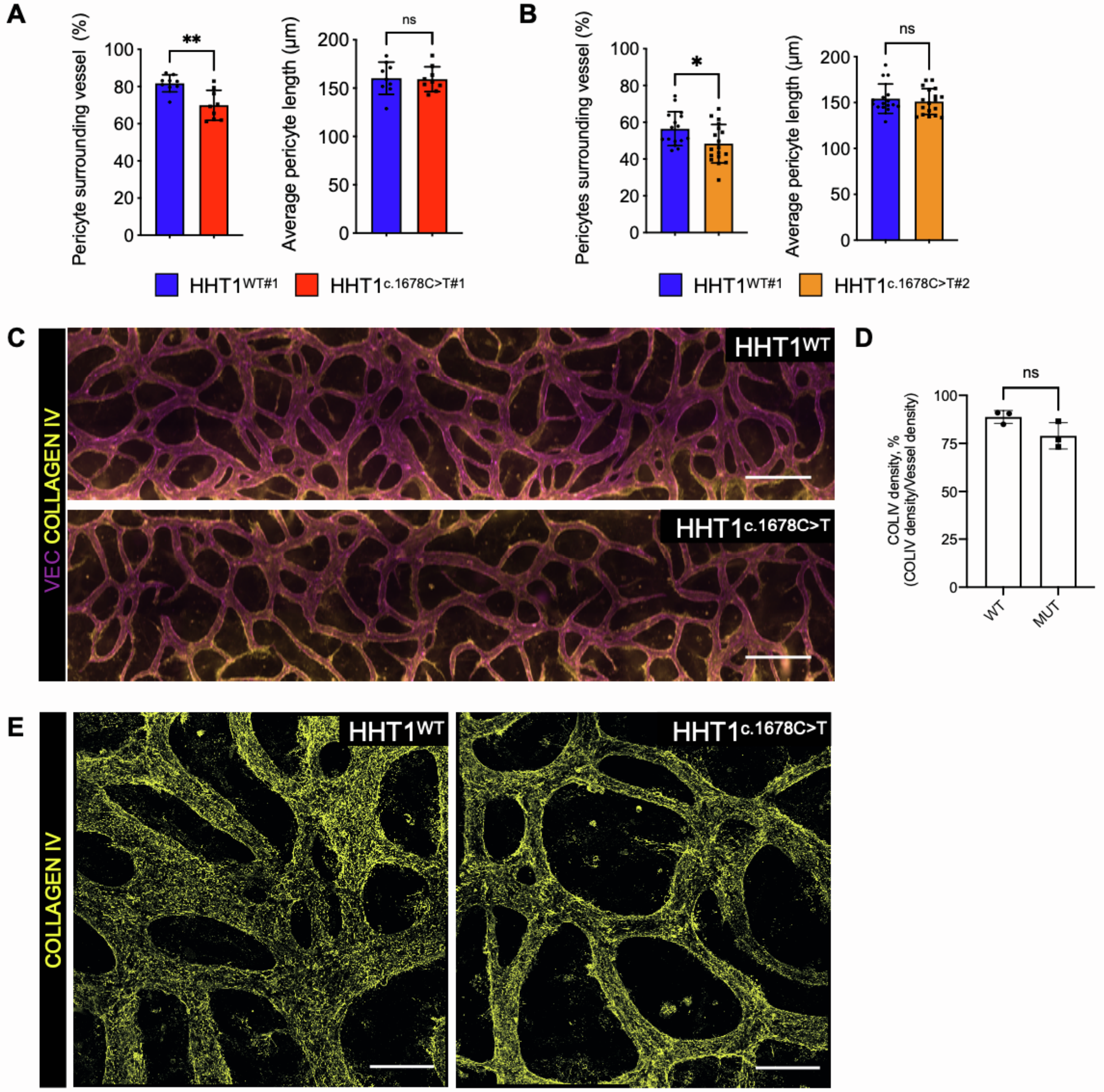

Figure S4. Related to Figure 3. HHT1-hiPSC-ECs show defective EC-pericyte cell interaction in VoC. (A, B) Quantification of % pericytes surrounding vessel and average length of pericytes using CellProfiler. From N=3, n=9; three independent biological experiments with mean from triplicate microfluidic channels per experiment (A). From N=5, n=15; five independent biological experiments with mean from triplicate microfluidic channels per experiment (B). Error bars are ±SD, Unpaired t-test. * $p<0.05$. (C) Representative images of vascular networks formed by HHT1-hiPSC-ECs differentiated from $HHT1^{WT}$ and $HHT1^{c.1678C>T}$ hiPSC lines in microfluidic chips. ECs are stained with anti-VEC (magenta) and anti-COLIV (yellow). Scale bar represents 500 µm. (D) Quantification of collagen IV density (%). Data are shown as ±SD from N=1, n = 3; one independent biological experiment, three microfluidic channels. Unpaired t-test. ns = not significant. (E) Representative spinning disk confocal images showing HHT1-hiPSC-EC vascular network stained for ECM (yellow; COLLAGEN IV). Scale bar 30 µm.

Supplemental Tables

Supplemental Table 1. List of FACS antibodies

Antibody	Fluorochrome	Source	Dilution	Catalog #
VE-Cadherin	A488	eBiosciences	1:50	53-1449-42
CD31	APC	eBiosciences	1:100	17-0319-42
KDR	PE	R&D systems	1:20	FAB357P
ENG	VioBlue	Miltenyi Biotec	1:20	130-099-666
ENG	PE-Vio-770	Miltenyi Biotec	1:20	130-099-889

Supplemental Table 2. List of antibodies for IF

Antibody	Species	Source	Dilution	Catalog #
SSEA-4	Mouse	Biolegend	1:30	330402
Nanog	Mouse	Santa Cruz	1:150	sc-293121
OCT3/4	Mouse	Santa Cruz	1:100	sc-5279
AFP	Rabbit	Quartett	1:25	2011200530
CD31	Mouse	Dako	1:200	M0823
βIII-tubulin	Mouse	Covance	1:4000	MMS-435P
VEC	Rabbit	CellSignaling	1:200	2158S
vWF	Rabbit	Dako	1:200	A0082
SOX17	Goat	R&D systems	1:200	AF1924
SM22	Rabbit	Abcam	1:200	AB14106
Collagen IV	Goat	Sigma	1:200	AB769
Laminin	Rabbit	Merck	1:100	AB19012
Alexa Fluor 488	Mouse IgG	Invitrogen	1:500	A11001
Alexa Fluor 568	Mouse IgM	Invitrogen	1:200	A21043
Alexa Fluor 488	Mouse IgG3	Invitrogen	1:250	A21151
Alexa Fluor 568	Mouse IgG1	Invitrogen	1:250	A21124
Alexa Fluor 647	Mouse IgG2b	Invitrogen	1:250	A21242
Alexa Fluor 568	Mouse IgG	Invitrogen	1:500	A11031
Alexa Fluor 488	Rabbit IgG	Invitrogen	1:500	A21206

Supplemental Experimental Procedures

Sanger sequencing
ENG mutations were confirmed by Sanger sequencing. DNA was extracted using the Gentra Puregene Cell Kit (QIAGEN) according to the manufacturer's protocol. PCR was performed to amplify *ENG* exon 12 (FW: CCAGAGTCAGGAGGGAGACA; RV: GCGTCCAGGATAGATTGCCT, Product size: 974 bp). The PCR products were purified using the QIAquick PCR Purification Kit and were send for Sanger sequencing (BaseClear).

Differentiation of hiPSC-ECs
hiPSC differentiation towards endothelial cells and CD31 magnetic bead isolation were performed as described previously (Halaidych et al., 2018; Orlova et al., 2014a, 2014b). Briefly, hiPSCs were passaged in normal culture conditions one day before inducing differentiation. Mesoderm differentiation was induced by changing the media to B(P)EL with a high concentration CHIR99021 (8 μM). At day 3, 6 and 9 of

differentiation the cells were refreshed with B(P)EL with VEGF (50 ng/ml) and SB43152 (10 μM, Tocris). ECs were purified with CD31 coupled magnetic beads at day 10 (Life Technologies) and the culture was further scaled up on 0,1% gelatin coated tissue culture flasks in human endothelial serum free media (EC-SFM)(Life Technologies) with additional VEGF (30 ng/ml), bFGF (20 ng/ml, R&D) and 1% platelet poor serum (PPS)(Hycultec). Functional assays were performed on cells between passages 2-3.

FACS analysis of hiPSC-ECs
FACS analysis was performed as described previously (Halaidych et al., 2018; Orlova et al., 2014a, 2014b). Purified hiPSC-ECs were dissociated with TrypLE Select (Life Technologies). Combinations of the following antibodies were used in flow cytometry experiments (see supplemental table 1): VE-Cadherin-A488 (1:50, eBiosciences), CD31-APC (1:100, eBiosciences), KDR-PE (1:20, R&D Systems), ENG-VioBlue or PE-Vio-770 (1:20, Milteny Biotec). Samples were analysed either on LSRII (BD) with the following instrument setup: Blue/488 FITC, A488: 505LP-530/30, PerCP-Cy5.5: 630LP-670/14; Yellow/561 PE: 570LP-582/15, APC: 635LP-660/20; or on MACSQuant VYB (Miltenyi) with the following instrument setup: Blue/488 FITC, A488: 525/50; Yellow/561 PE: 586/15, APC: 661/20, APC-Cy7: 750LP.

Immunofluorescent staining of hiPSC-ECs
Immunofluorescent staining was performed as described previously (Halaidych et al., 2018; Orlova et al., 2014a, 2014b). Briefly, ECs were seeded on FN-coated 96-well black imaging plates (Corning) at the seeding density ~10,000cells/well in complete EC growth culture medium (EC-SFM supplemented with VEGF 30 ng/ml, bFGF 20 ng/ml and 1% PPS). 48h post-seeding ECs were fixed with 4% paraformaldehyde (PFA, Sigma), permeabilized with the 0.1%TX-100 and stained with anti-CD31 (1:200, Dako) and anti-vWF (1:200, Dako) or VEC (CellSignaling)(supplemental table 2). High magnification images were acquired with the WLL1 confocal microscope (Leica), using 40x DRY objective.

Endothelial cell proliferation (MTS assay)
hiPSC-ECs were seeded into on FN-coated 96-well plates at the seeding density 2,000 cells/well in EC-SFM for 12 h and subsequently refreshed with EC-SFM containing various stimuli. After 4 days the MTS assay (CellTiter, Promega) was used to determine the relative number of ECs.

Assessment of hiPSC-ECs functionality in an *in vitro* vasculogenesis assay
The co-culture experiments with hiPSC-ECs or primary ECs and stromal cells were performed as described previously (Halaidych et al., 2018; Orlova et al., 2014a, 2014b). The co-cultures were stopped at day 10 and post-fixed and stained with anti-CD31 (1:200, Dako) and anti-SOX17 (1:200, R&D), and anti-SM22 (1:200, Abcam) antibodies (supplemental table 2). The co-cultures were imaged with the EVOS FL AUTO2 Imaging system (ThermoFischer Scientific) with the 10X Objective for quantifications with autofocus on CD31, and auto stitching 4X4 focus planes or 20X Objective for CD31 and SOX17 images. The co-cultures were quantified using publicly available software AngioTool (Zudaire et al., 2011).

Endothelial barrier function analysis
Endothelial barrier function was measured using impedance-based cell monitoring with an electric cell-substrate impedance sensing system (ECIS Zθ, Applied Biophysics), as described previously (Halaidych et al., 2018). hiPSC-ECs were seeded on FN-coated ECIS arrays each containing 8 wells with 10 gold electrodes per well (8W10E PET, Applied Biophysics). The cell seeding density was estimated ~50,000cells/cm^2. For barrier function and migration studies the cells were seeded for at least 24h in complete EC growth medium followed by 6h serum starvation step in EC-SFM. For the assessment of cell migration after serum starvation, the medium was changed to complete EC growth medium or EC-SFM supplemented with 1% PPS, BMP9 (2ng/ml) and TGFβ3 (1ng/ml), and electric wound (10 sec pulse of 5V at 60 kHz) was applied to the cells 1h after medium change. Recovery of the barrier was monitored in real time over 6-12h. Multiple frequency/time (MFT) mode was used for the real-time assessment of the barrier and monolayer confluence.

Generation of perfused vascular networks in microfluidic chips
Vascular networks were generated as described previously (Chen et al., 2017) with some adjustments that were developed during optimization of the protocol with hiPSC-ECs and primary human brain vascular pericytes (HBVPs)(ScienceCell), and microfluidic chips with one gel channel and two media channels (AIM

Biotech). Cells were resuspended in EGM-2 supplemented with thrombin (4 U/ml) at $10x10^6$ cells/ml for hiPSC-ECs and $0.5x10^6$ cells/ml for HBVPs or $2x10^6$ cells/ml for HBVPs (note: higher HBVP numbers promote vessel formation, although earlier experiments were conducted with a lower number of HBVPs with comparable results). The cell suspension was mixed with an equal volume of fibrinogen solution (10 mg/ml; final concentration 5 mg/ml) and injected into the gel channel of the microfluidic chip; this t was left for 15 min at room temperature (RT) to allow fibrin gel to form. EGM-2 supplemented with VEGF (50 ng/ml) was added to each of the flanking media channels. Interstitial flow through the gel was achieved by adding a larger volume of medium to one of the media inlets, generating a pressure gradient. The microfluidic chips were refreshed every 24 hours with EGM-2 supplemented with VEGF (50 ng/ml) and γ-secretase inhibitor N-[N-(3,5-difluorophenacetyl)-l-alanyl]-s-phenylglycinet-butyl ester (DAPT, 10 μM) (DAPT supplementation was performed on day 1 for 24 hours). For immunofluorescent staining, 3D cultures were fixed with 4% paraformaldehyde (PFA; Sigma) for 20 min at RT, permeabilized with 0.5% TX-100 for 15 min at RT and blocked with 3% bovine serum albumin (BSA) in PBS for 3 hours at RT. Samples were stained by anti-CD31 (1:200, Dako), anti-SOX17(1:200, R&D), and anti-SM22 (1:200, Abcam). Details of antibodies are given in supplemental table 2. Primary antibodies were prepared in 2% BSA and incubated overnight at 4°C and secondary antibodies were prepared in 2% BSA and incubated for 2 hours at RT. Fluorescence images for quantification were acquired using EVOS AUTO2 using 10x magnification objective and high magnification images were acquired with a DragonFly spinning disk (Andor) microscope using 40x magnification objective post-processing, performed and processed using Imaris 9.5 software (Bitplane, Oxford Instruments).

EdU assay for EC proliferation in 3D microfluidic chips

Proliferation was measured using an EdU Click-iT kit Alexa-488 (ThermoFisher Scientific #C10337) according to manufacturer's protocol. Briefly, on day 6 of culture, microfluidic chips were refreshed with EGM-2 (VEGF 50 ng/ml and DAPT 10 μM) additionally supplemented with EdU (1:1000) for 8 hours. Cells were fixed with 4% PFA for 30 minutes, permeabilised with 0.5% TX-100 for 15 minutes at RT. Freshly prepared Click-iT reaction cocktail was added for 30 minutes at RT. Microfluidic chips were washed twice with 3% BSA-PBS and blocked in 3% BSA-PBS for 1-2 hours at RT, followed by co-staining with primary and secondary antibodies.

Quantification of 3D vessels in microfluidic chips

Microfluidic chips were imaged with the EVOS FL AUTO2 or M7000 Imaging system (ThermoFischer Scientific) using the 10X Objective with autofocus either on the cells in phase-contrast mode (for time series experiments) or on CD31 for fixed samples, and automatic image stitching to cover the entire gel channel for quantification of microvascular networks. Parameters were quantified using pipelines developed on the free open source CellProfiler software (https://cellprofiler.org/) (Carpenter et al., 2006). Briefly, for EC nuclei number, pre-processing steps were used to enhance image features and filter non-specific object identification. A Gaussian filter was applied to mural cell images before object identification was used to measure object morphology and interaction with hiPSC-EC network. Filter steps were applied to images of vascular network to reduce non-specific segmentation from cell junction staining and a minimum cross-entropy thresholding method was used to produce a binarized image. The binarized images from the CellProfiler output were then analysed using freely available ImageJ software with the plugin (https://imagej.nih.gov/ij/, https://imagej.net/DiameterJ) (Hotaling et al., 2015). For ECM quantification, binarized vessel images were used as masks to ensure Collagen IV staining intensity was quantified only at the vessel regions excluding any background noise. The number of EdU and SOX17 positive nuclei were quantified with a custom-made pipeline in CellProfiler (Carpenter et al., 2006). Additional quantification of the distance between SM22- and CD31 positive cells was obtained using Imaris 9.5 software (Bitplane, Oxford Instruments).

Perfusion assessment in vessels in microfluidic chips

For perfusion assessment, the chip was placed on day 6 in the EVOS AUTO2 with on stage incubator for time-lapse image acquisition. First, basal fluorescence activity was captured before the addition of fluorescent tracers. Next, 70 µl of 40KDa FITC-Dextran (1:1000, Sigma) or 405-beads (1:10, Fluoro-Max Dyed Blue Aqueous Fluorescent Particles, B0200, ThermoFisher Scientific) in EGM-2 was added to one medium port and 50 µl of EGM-2 to all other media ports to induce interstitial gravity flow. Then, images were captured simultaneously at 20 fps using a 10x magnification objective for 30 seconds. For

quantification of permeability coefficient, images acquired at T1 (time 0 seconds) and T2 (30 seconds of perfusion) were quantified using pipelines developed with CellProfiler (Carpenter et al., 2006). Briefly, a mask for vessel area inside image acquired was created to ensure intensity was measured in area outside the vessel wall only. Calculation for permeability coefficient was based on the previously established method (Campisi et al., 2018).

Supplemental References

Campisi, M., Shin, Y., Osaki, T., Hajal, C., Chiono, V., and Kamm, R.D. (2018). 3D self-organized microvascular model of the human blood-brain barrier with endothelial cells, pericytes and astrocytes. Biomaterials *180*, 117–129.

Carpenter, A.E., Jones, T.R., Lamprecht, M.R., Clarke, C., Kang, I.H., Friman, O., Guertin, D.A., Chang, J.H., Lindquist, R.A., Moffat, J., et al. (2006). CellProfiler: image analysis software for identifying and quantifying cell phenotypes. Genome Biology *7*, R100.

Chen, M.B., Whisler, J.A., se, J.F. ouml, Yu, C., Shin, Y., and Kamm, R.D. (2017). On-chip human microvasculature assay for visualization and quantification of tumor cell extravasation dynamics. Nature Protocols *12*, 865–880.

Halaidych, O.V., Freund, C., Hil, F. van den, Salvatori, D.C.F., Riminucci, M., Mummery, C.L., and Orlova, V.V. (2018). Inflammatory Responses and Barrier Function of Endothelial Cells Derived from Human Induced Pluripotent Stem Cells. Stem Cell Reports *10*, 1642–1656.

Hotaling, N.A., Bharti, K., Kriel, H., and Simon, C.G. (2015). DiameterJ: A validated open source nanofiber diameter measurement tool. Biomaterials *61*, 327–338.

Orlova, V.V., Drabsch, Y., Freund, C., Petrus-Reurer, S., Hil, F.E. van den, Muenthaisong, S., Dijke, P. ten, and Mummery, C.L. (2014a). Functionality of endothelial cells and pericytes from human pluripotent stem cells demonstrated in cultured vascular plexus and zebrafish xenografts. Arteriosclerosis, Thrombosis, and Vascular Biology *34*, 177–186.

Orlova, V.V., Hil, F.E. van den, Petrus-Reurer, S., Drabsch, Y., Dijke, P. ten, and Mummery, C.L. (2014b). Generation, expansion and functional analysis of endothelial cells and pericytes derived from human pluripotent stem cells. Nature Protocols *9*, 1514–1531.

Zudaire, E., Gambardella, L., Kurcz, C., and Vermeren, S. (2011). A Computational Tool for Quantitative Analysis of Vascular Networks. PLoS ONE *6*, e27385-12.
