# Supplementary material for: Vascular defects associated with hereditary hemorrhagic telangiectasia revealed in patient-derived isogenic iPSCs in 3D vessels on chip
Source: Stem Cell Reports. 2022 Jun 30;17(7):1536–45. doi: 10.1016/j.stemcr.2022.05.022 (PMC9287680; doi:10.1016/j.stemcr.2022.05.022)
Supplement: Document S2. Article plus supplemental information [file mmc4.pdf]

Stem Cell Reports

Report

OPEN ACCESS

Vascular defects associated with hereditary hemorrhagic telangiectasia revealed in patient-derived isogenic iPSCs in 3D vessels on chip

Valeria V. Orlova,[1,*] Dennis M. Nahon,[1] Amy Cochrane,[1] Xu Cao,[1] Christian Freund,[2] Francijna van den Hil,[1] Cornelius J.J. Westermann,[3] Repke J. Snijder,[3] Johannes Kristian Ploos van Amstel,[4] Peter ten Dijke,[5] Franck Lebrin,[6,7] Hans-Jurgen Mager,[3] and Christine L. Mummery[1,2,*]

[1]Department of Anatomy and Embryology, Leiden University Medical Center, Leiden 2333ZA, the Netherlands
[2]Department of Anatomy and Embryology and Human iPSC Hotel, Leiden University Medical Center, Leiden 2333ZA, the Netherlands
[3]St. Antonius Hospital, Nieuwegein, the Netherlands
[4]Department of Genetics, University Medical Center, Utrecht, the Netherlands
[5]Department of Cell and Chemical Biology, Oncode Institute, Leiden University Medical Center, Leiden 2333ZA, the Netherlands
[6]Einthoven Laboratory for Experimental Vascular Medicine, Department of Internal Medicine, Leiden University Medical Center, Leiden, the Netherlands
[7]INSERM U1273, ESPCI, CNRS FRE 2031, Paris, France
*Correspondence: v.orlova@lumc.nl (V.V.O.), c.l.mummery@lumc.nl (C.L.M.)
https://doi.org/10.1016/j.stemcr.2022.05.022

SUMMARY

Hereditary hemorrhagic telangiectasia (HHT) is a genetic disease characterized by weak blood vessels. HHT1 is caused by mutations in the *ENDOGLIN* (*ENG*) gene. Here, we generated induced pluripotent stem cells (hiPSCs) from a patient with rare mosaic HHT1 with tissues containing both mutant (*ENG*$^{c.1678C>T}$) and normal cells, enabling derivation of isogenic diseased and healthy hiPSCs, respectively. We showed reduced ENG expression in HHT1 endothelial cells (HHT1-hiPSC-ECs), reflecting haploinsufficiency. HHT1$^{c.1678C>T}$-hiPSC-ECs and the healthy isogenic control behaved similarly in two-dimensional (2D) culture, forming functionally indistinguishable vascular networks. However, when grown in 3D organ-on-chip devices under microfluidic flow, lumenized vessels formed in which defective vascular organization was evident: interaction between inner ECs and surrounding pericytes was decreased, and there was evidence for vascular leakage. Organs on chip thus revealed features of HHT in hiPSC-derived blood vessels that were not evident in conventional 2D assays.

INTRODUCTION

Hereditary hemorrhagic telangiectasia (HHT) is an inherited genetic disorder caused by autosomal dominant mutations in Endoglin (*ENG*; HHT1), Activin receptor like kinase-1 (*ACVRL1*; HHT2) or *SMAD4* (HHT3), genes that mediate signaling by transforming growth factor β (TGF-β) and bone morphogenetic protein (BMP) in vascular endothelial cells (ECs) (Goumans et al., 2009). Phenotypically, HHT causes tortuous defects in blood vessels, particularly evident in the skin and mucous membranes, that are prone to hemorrhage (Govani and Shovlin, 2009). These abnormalities, called telangiectases, consist of enlarged and dilated capillaries that lack the pericyte/smooth muscle cell coverage of normal vessels. Studies in mice indicated that ENG deficiency can lead to abnormal endothelial-pericyte cell interactions caused by defective paracrine signaling by ECs lacking ENG (Carvalho et al., 2004; Lebrin et al., 2010). More severe abnormalities, evident as arteriovenous malformations (AVMs) in the brain, lung, liver, and gastrointestinal tract, can be fatal if hemorrhage occurs (Govani and Shovlin, 2009). To date, there are no therapies that prevent the formation of these abnormalities in patients with HHT or reverse them once they have occurred. At most, current therapies, such as surgical intervention or cauterization of vessels to divert blood flow, ameliorate symptoms of the disease but are not cures (Shovlin, 2010). Medical treatments under investigation include anti-angiogenic, -inflammatory, and -fungal drugs such as humanized anti-vascular endothelial growth factor (VEGF) antibody (bevacizumab) (Dupuis-Girod et al., 2012), thalidomide (Lebrin et al., 2010), itraconazole (Kroon et al., 2020), and other drugs (reviewed in Snodgrass et al., 2021). Genetic models of HHT in mice, in which the genes responsible for the disease in humans are deleted, show clear vascular defects, but they have not shed much light on the specific genotype/phenotype relationships in HHT patients (Tual-Chalot et al., 2015). Attempts to model HHT using primary human umbilical vein ECs (HUVECs) isolated from newborn HHT patients failed to recapitulate the phenotype (Chan et al., 2004). Blood outgrowth ECs (BOECs) or peripheral blood monocytes (PBMCs) from patients with HHT could be alternative sources of cells to model HHT (Begbie et al., 2003; Fernandez-L et al., 2005; Laake et al., 2006), but their poor proliferation *in vitro* makes them unsuitable as a renewable source of ECs for reproducibly modeling the disease in humans and for drug discovery.

In the present study, we aimed to establish an efficient and scalable system that would recapitulate the formation of defective blood vessels in patients with HHT, based on patient-derived human induced pluripotent stem cells (HHT1-hiPSCs). We hypothesized that HHT1-hiPSCs might be useful for (1) identifying mechanisms underlying disease predisposition and modeling clinical features of

 © 2022 The Author(s).
This is an open access article under the CC BY license (http://creativecommons.org/licenses/by/4.0/).

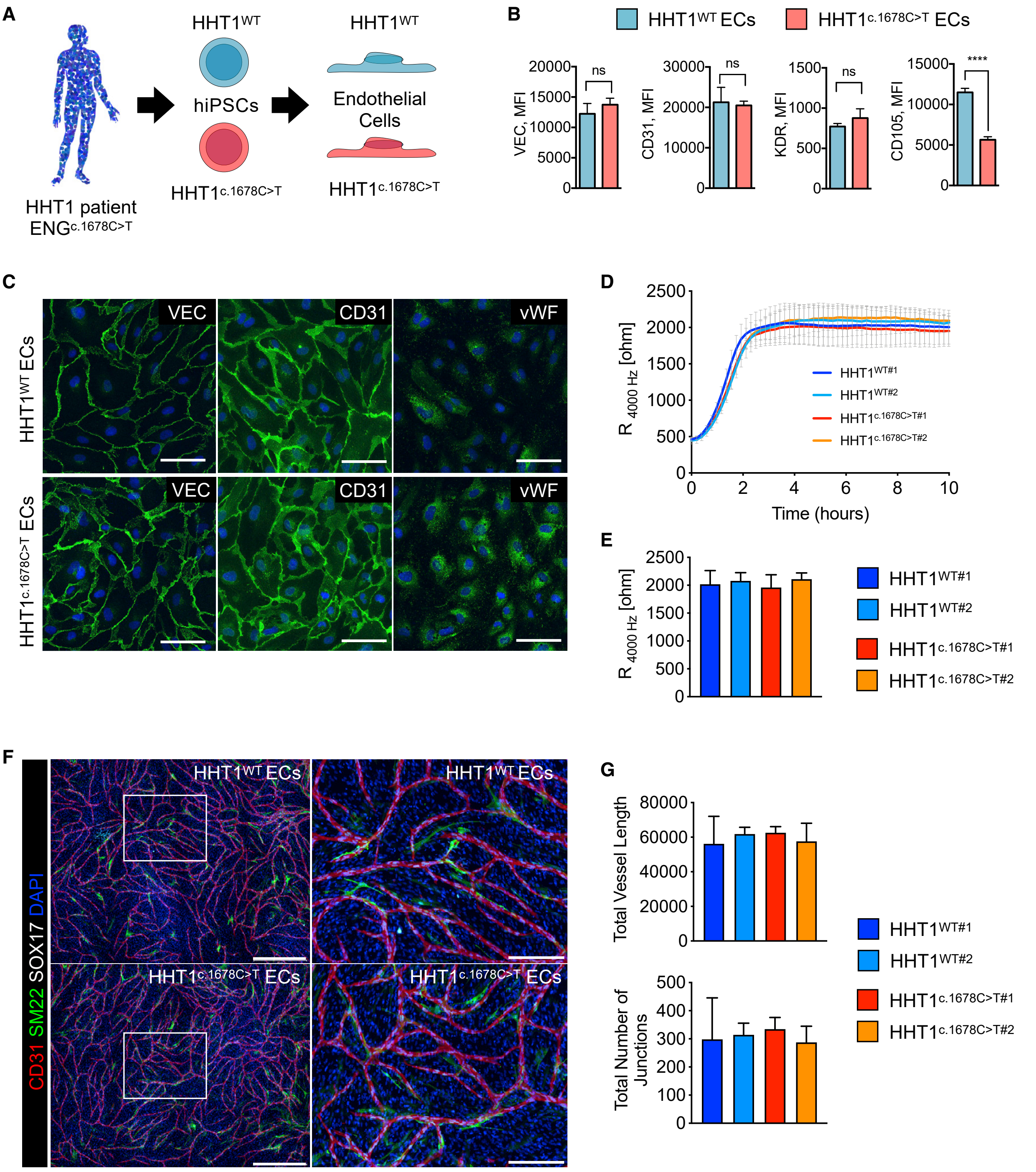

Figure 1. Characterization of HHT1-hiPSC-ECs

(A) Schematic overview of ECs differentiated from HHT1 patient-derived isogenic hiPSCs.

(B) FACS analysis of surface VEC, CD31, KDR, and ENG (CD105) expression on isolated ECs (P2) from HHT1^{WT} and HHT1$^{c.1678C>T}$ hiPSC lines. ECs differentiated from three independent hiPSC clones were analyzed. Median fluorescent intensity values are shown. Error bars are ± SD. Unpaired t test. ****p < 0.001.

(legend continued on next page)

HHT1 *in vitro* and (2) investigating defective endothelial-pericyte interactions.

RESULTS

HHT1-hiPSC-ECs reflect ENG haploinsufficiency with no apparent differences in functionality

hiPSC lines were generated from somatic tissue from a patient with HHT1 with a heterozygous nonsense mutation in *ENG* (NM_001114753.2 (*ENG*):c.1678C>T; p.(Gln560*)), which causes ENG haploinsufficiency (Letteboer et al., 2005). The patient was identified as being a genetic mosaic, allowing generation of isogenic pairs of hiPSC lines with and without the mutation ($HHT1^{c.1678C>T}$ and $HHT1^{WT}$) (Figures S1A–S1C; unpublished data). HHT1-hiPSC clones had normal karyotypes and were verified as pluripotent using standard methods (PluriTest, expression of pluripotency markers and spontaneous differentiation toward three germ cell lineages) (Figures S1D–S1G). HHT1-hiPSCs were then induced to differentiate to ECs (Orlova et al., 2014a; 2014b). Surface ENG (CD105) was significantly downregulated in $HHT1^{c.1678C>T}$-hiPSC-ECs compared with $HHT1^{WT}$-hiPSC-ECs (Figures 1A and 1B). By contrast, surface expression of other major EC markers such as vascular endothelial cadherin (VEC), platelet and endothelial adhesion molecule (CD31/PECAM1), and kinase insert domain receptor (KDR), also known as VEGFR2, was similar among lines (Figure 1B). ENG haploinsufficiency had no apparent effect on the proliferation of HHT1-hiPSC-ECs (Figure S2A). HHT1-hiPSC-ECs showed similar responses upon short-term stimulation with BMP9 and TGF-β, except that *ID1* expression was significantly upregulated in $HHT1^{c.1678C>T}$-hiPSC-ECs after 2 h of TGF-β treatment (Figure S2B).

Immunostaining showed comparable expression and localization of VEC, CD31, and intracellular von Willebrand factor (vWF) (Figure 1C). Barrier function was assessed by real-time impedance spectroscopy (electric cell-substrate impedance sensing [ECIS]) in an integrated assay of electric wound healing for migration, as previously described (Halaidych et al., 2018). Barrier function in two dimension (2D) was similar in healthy and HHT1-hiPSC-ECs in "complete" EC growth medium (CGM) (Figures 1D and 1E), and migration rates were identical (data not shown). Barrier function was increased in growth-factor-free medium (1% platelet-poor plasma serum [PPS]) (Figures S2C and S2D), in line with our previous findings (Halaidych et al., 2018). BMP9 addition reduced barrier function equally in healthy and $HHT1^{c.1678C>T}$-hiPSC-ECs (Figures S2C and S2D). TGF-β addition significantly reduced barrier function in $HHT1^{c.1678C>T}$-hiPSC-ECs but had no significant effect in $HHT1^{WT}$-hiPSC-ECs (Figures S2C and S2D), which is also in line with the differences in responses in HHT1-hiPSC-ECs upon TGF-β treatment. Migration rates were similar in all conditions examined (Figure S2E).

Finally, the ability to form 2D vascular networks *in vitro* was examined, as described previously (Halaidych et al., 2018; Orlova et al., 2014a, 2014b). HHT1-hiPSC-ECs formed well-organized vascular networks when cultured with stromal cells, and these were indistinguishable from healthy controls. Furthermore, stromal cells adjacent to ECs upregulated expression of the contractile smooth muscle cell marker SM22 (Figure 1F). Quantification of vascular networks formed by $HHT1^{c.1678C>T}$-hiPSC-ECs and $HHT1^{WT}$-hiPSC-ECs showed similar total vessel length and number of branches (Figure 1G) as well as total number of $SOX17^{+}$ ECs and number of adjacent $SM22^{+}$ cells (data not shown).

HHT1-hiPSC-ECs show defective vascular organization in 3D microfluidic chips

The ability of HHT1-hiPSC-ECs to form microvascular networks in a 3D vessel-on-chip (VoC) model was then examined (Figure 2A). Primary human brain vascular pericytes (HBVPs) were used to support microvascular-network formation, as described previously (Vila Cuenca et al., 2021). Vascular networks developed around day 2–3 of culture; lumenized microvessels were observed around day 5 (Figure S3A). Microfluidic chips were immunostained with

(C) Immunofluorescent analysis of EC markers VEC, CD31, and vWF on isolated ECs from HHT1 patient-derived isogenic hiPSCs (P2). Scale bar: 75μm.

(D) Absolute resistance of the EC monolayer at 4,000 Hz in complete EC growth medium (CGM). ECs differentiated from two independent hiPSC clones were analyzed. Error bars are ± SD of three to five independent biological experiments per clone.

(E) Quantification of absolute resistance values at 4,000 Hz from (D). Error bars are shown as ± SD of five independent biological experiments.

(F) Representative immunofluorescent images of an *in vitro* vasculogenesis sprouting assay at day 10 of the co-culture of ECs differentiated from two independent clones of HHT1 patient-derived isogenic hiPSCs and $CD31^{-}$ cells differentiated from an independent control hiPSC line. ECs are stained with anti-CD31 (red) and anti-SOX17 (gray), contractile $CD31^{-}$ cells with anti-SM22 (green), and nuclei with DAPI (blue). Left panels: automatically stitched images (10× objective, 4 × 4 focus planes) are shown; scale bar: 750 μm. Right panels: magnification of the framed area in the left panel. Scale bar: 250 μm.

(G) Quantification of EC sprouting network at day 10 of the co-culture. The total vessel length and total number of junctions are shown. Data are shown as ± SD of three independent biological experiments.

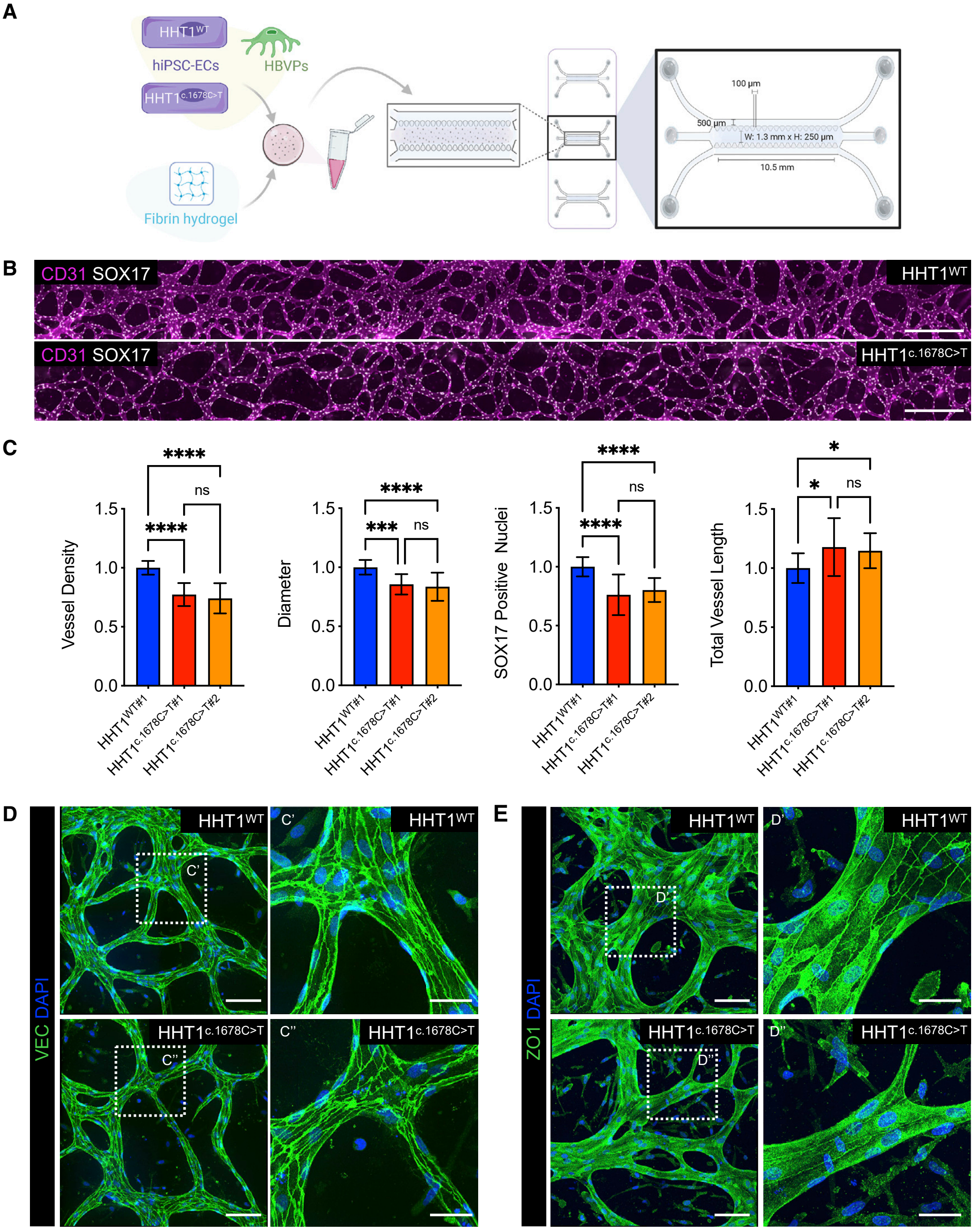

Figure 2. HHT1-hiPSC-ECs show defective vascular organization in 3D microfluidic chips

(A) Schematic showing generation of 3D vascular networks in microfluidic chips using hiPSC-ECs and HBVPs.

(B) Representative images of vascular networks formed by HHT1-hiPSC-ECs differentiated from HHT1^{WT} and HHT1$^{c.1678C>T}$ hiPSC lines in microfluidic chips. ECs are stained with anti-CD31 (magenta) and anti-SOX17 (yellow). Scale bar: 500 μm.

(C) Quantification of HHT1-hiPSC-EC vascular network showing vessel density, diameter, number of HHT1-hiPSC-ECs (SOX17+ nuclei), and total vessel length. Data are shown as ± SD, Unpaired t test. ****$p < 0.0001$, ***$p < 0.0005$, *$p < 0.05$, ns, not significant. Normalized

(legend continued on next page)

antibodies against CD31/PECAM1 and an EC-specific transcription factor SOX17. The ability to form microvascular networks in microfluidic chips was compromised in HHT1$^{c.1678C>T}$-hiPSC-ECs compared with HHT1^{WT}-hiPSC-ECs (Figures 2B and 2C), despite similar initial seeding densities (Figure S3A). Quantification of microvascular networks showed reduced vascular density, diameter of the vessels, and number of ECs (SOX17+ nuclei) and an increase of the total length of the vessels of the networks formed by HHT1$^{c.1678C>T}$-hiPSC-ECs (Figures 2C, S3C and S3D). HHT1^{WT}-hiPSC-ECs from two independent hiPSC clones behaved similarly (Figure S3B). Furthermore, proliferation of HHT1$^{c.1678C>T}$-hiPSC-ECs was lower than HHT1^{WT}-hiPSC-ECs, as evidenced by fewer EdU-positive nuclei (Figures S3E and S3F). To demonstrate that the microvascular networks formed by HHT1-hiPSC-ECs were hollow, fluorescent beads were perfused through the vessels (Video S1). Notably, the beads moved at a considerably lower rate in the microvascular networks formed by HHT1$^{c.1678C>T}$-hiPSC-ECs compared with HHT1^{WT}-hiPSC-ECs, indicating reduced flow through 3D vessels formed by HHT1$^{c.1678C>T}$-hiPSC-ECs.

Junctional integrity was examined by immunostaining of the microvascular networks with VEC and ZO1 (Figures 2D and 2E). The results showed that although VEC junctional distribution was comparable, junctional distribution of ZO1 was reduced in the microvascular networks formed by HHT1$^{c.1678C>T}$-hiPSC-ECs compared with HHT1^{WT}-hiPSC-ECs.

HHT1-hiPSC-ECs show defective EC-pericyte interaction

We next analyzed the interaction between HHT1-hiPSC-ECs and HBVPs in the VoC model. Microfluidic chips were immunostained with antibodies against CD31/PECAM1 and the contractile smooth muscle cell marker SM22 (Figure 3A). Quantification of microvascular networks formed by HHT1$^{c.1678C>T}$-hiPSC-ECs showed reduced pericyte coverage compared with HHT1^{WT}-hiPSC-ECs (Figures 3B, S4A, and S4B). HHT1$^{c.1678C>T}$-hiPSC-ECs also showed increased pericyte distance from ECs when compared with HHT1^{WT}-hiPSC-ECs (Figures 3C and 3D). Surface rendering of confocal images revealed that pericytes in vascular segments formed by HHT1$^{c.1678C>T}$-hiPSC-ECs were positioned more distally from ECs compared with vascular segments formed by HHT1^{WT}-hiPSC-ECs (Figures 3E and 3F). No significant differences were found in extracellular matrix (ECM) organization in microvascular networks formed by HHT1^{WT}-hiPSC-ECs and HHT1$^{c.1678C>T}$-hiPSC-ECs, as demonstrated by counterstaining with VEC and collagen IV (COLIV) (Figures S4C–S4E).

We next performed a fluorescent dextran leakage assay to test whether reduced EC-pericyte interaction caused the 3D vessels to be more fragile and prone to leak. Fluorescently labeled dextran (FITC-Dextran, 40 kDa) was added into the medium channel of the organ-on-chip device, and real-time videos of vascular segments pre-stained using fluorescent agglutinin were made (Figure 4A; Video S2). Quantification of permeability coefficient showed increased leakage of 3D vascular segments formed from HHT1$^{c.1678C>T}$-hiPSC-ECs compared with HHT1^{WT}-hiPSC-ECs (Figures 4B and 4C).

DISCUSSION

In this study, we developed a human *in vitro* model for the genetic vascular disorder HHT using hiPSCs derived from patients with mutations in the *ENG* gene (HHT1). The results showed that we likely captured the direct effects of reduced ENG protein on the EC surface without compensation or adaption mechanisms that normally occur *in vivo,* notably in mutant mice (El-Brolosy and Stainier, 2017). Thus, even though differences in vessels were observed, aspects of the HHT phenotype were masked although the poor EC-pericyte interaction was similar to that reported previously in Eng$^{+/-}$ mutant mice (Galaris et al., 2019). This would contribute to vessel instability and could cause the leaky 3D vascular network formed by HHT1$^{c.1678C>T}$-hiPSC-ECs, in line with observations in patients.

The results in the HHT1-hiPSC-EC 2D model differ from earlier studies in which small interfering RNA (siRNA) was used to knock down *Eng* transiently in mouse embryonic ECs (Lebrin et al., 2004). Complete *Eng* knockdown in mouse embryonic ECs resulted in reduced EC proliferation and TGF-β signaling in 2D assays. On the other hand, *ENG* haploinsufficiency had no effect on EC function in 2D, with proliferation, barrier function, and sprouting angiogenesis in ECs derived from HHT1$^{c.1678C>T}$-hiPSC clones indistinguishable from isogenic controls.

To establish a HHT1-hiPSC-ECs VoC model, we used a commercially available microfluidic chip that supports formation of an interconnected microvascular network (Campisi et al., 2018; Chen et al., 2017; Shin et al., 2012; Vila Cuenca et al., 2021). The model allows simultaneous analysis of both the early steps of the 3D vascular-network

values from independent experiments are shown. From N = 3, n = 9; three independent experiments with three microfluidic channels per experiment (HHT$^{WT\#1}$, HHT$^{c.1678C>T\#1}$). From N = 5, n = 15; five independent experiments with three microfluidic channels per experiment (HHT$^{WT\#1}$, HHT$^{c.1678C>T\#2}$).
(D and E) Representative confocal images showing VEC (D) and ZO1 (E). Nuclei are stained with DAPI (blue). Inserts are magnifications of framed areas to show VEC and ZO1 localization. Scale bars: 100 μm (left panels) and 40 μm (right panels).

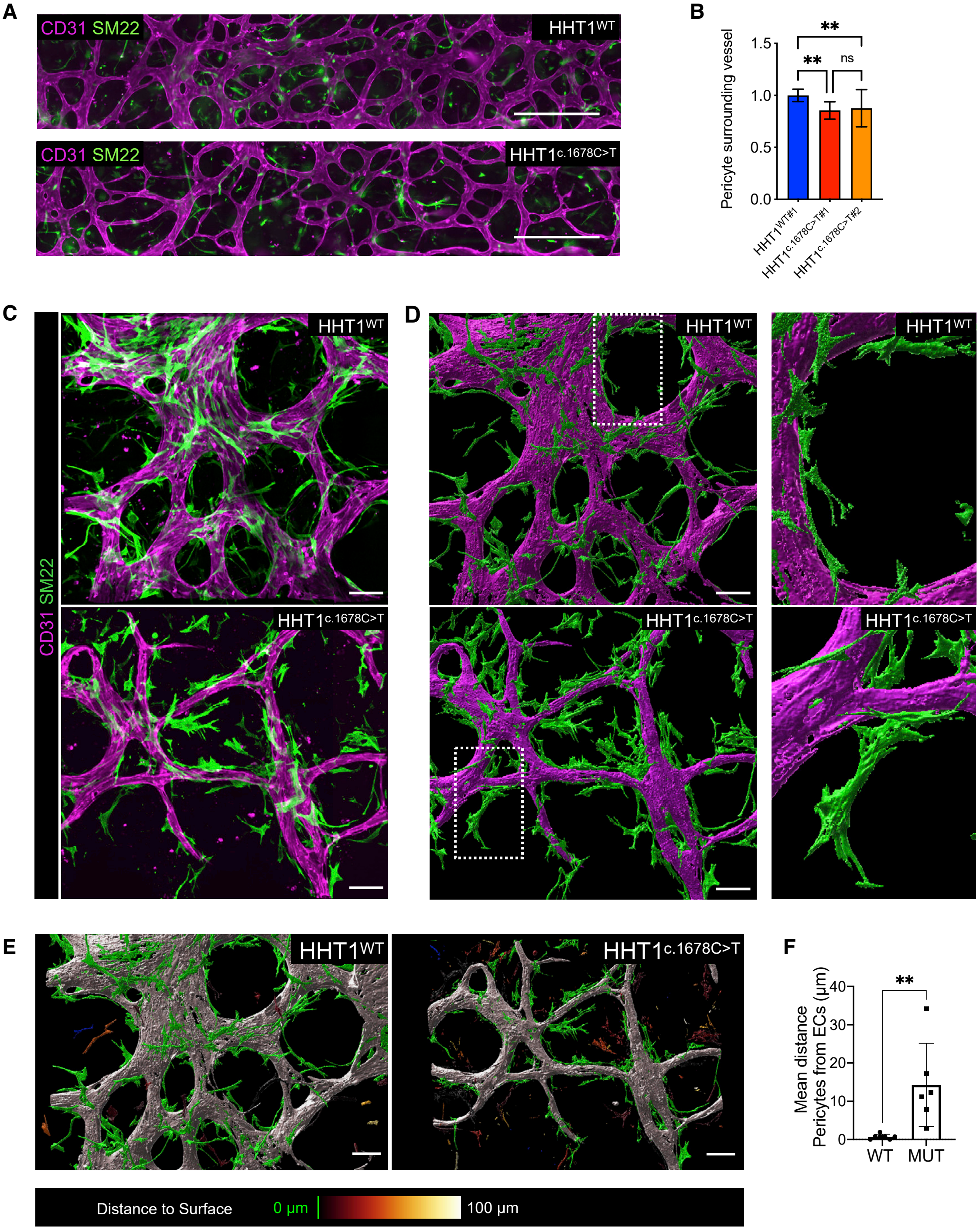

Figure 3. HHT1-hiPSC-ECs show defective EC-pericyte cell interaction

(A) Representative images of vascular networks formed by HHT1-hiPSC-ECs differentiated from HHT1^{WT} and HHT1$^{c.1678C>T}$ hiPSC lines in microfluidic chips. ECs are stained with anti-CD31 (magenta) and anti-SM22 (green). Scale bar: 500 μm.

(B) Quantification of percentage of pericytes surrounding vessel and average length of pericytes using CellProfiler. Normalized values from independent experiments are shown. From N = 3, n = 9; three independent experiments with three microfluidic channels per experiment

(legend continued on next page)

formation, such as EC cell proliferation, migration, lumen formation, remodeling and pruning (regression), and endpoint analysis. This includes high-resolution microscopy for EC-pericyte interaction, perfusion studies, and vascular leakage assays. Gravity-driven flow in these chips is sufficient for the maintenance of the vascular segments that are perfused with non-perfused vascular segments regressing overtime, similar to what was observed *in vivo* (Franco et al., 2015; Kochhan et al., 2013).

Overall, we found that HHT1$^{c.1678C>T}$-hiPSC-ECs showed multiple similarities to Eng$^{+/-}$ mutant mice (Arthur et al., 2000; Carvalho et al., 2004; Lebrin et al., 2010), although there were some differences. These included the formation of narrower vessels with fewer ECs by HHT1$^{c.1678C>T}$-hiPSC-ECs than healthy controls. Furthermore, HHT$^{c.1678C>T}$-hiPSC-ECs showed reduced junctional localization of ZO1, although localization of VEC was comparable. This could be a result of reduced EC-matrix adhesion, which in turn could affect cell-cell junctions (Yamamoto et al., 2015) and reduced perfusion and increased regression of vascular networks, as described previously in *eng* mutant zebrafish (Sugden et al., 2017), resulting in reduced EC-pericyte interaction. However, the role of ENG in regulation of cell-to-cell and cell-to-matrix adhesion is beyond the scope of the present study. Thus, despite some shortcomings of the VoC model in capturing the complete HHT patient phenotype, we believe the model is a valuable tool to investigate underlying causes of poor EC-pericyte interaction and identify drugs to reverse it and mediate vascular normalization.

Additional triggers of AVM formation include somatic mutations that reduce ENG function, local loss of ENG protein caused by inflammation, and pro-angiogenic triggers (Mahmoud et al., 2010; Tual-Chalot et al., 2015). Loss of ENG function in mutant mice was shown to induce defective migration against blood flow and EC enlargement, which caused vessels to dilate (Sugden et al., 2017). This, in turn, results in higher hemodynamic forces and peripheral hypoxia that support the enlargement of AVMs (Sugden and Siekmann, 2018). Our current model mainly addressed ENG haploinsufficiency due to *ENG* gene defects and lacked the additional triggers that cause ENG loss of function, such as exposure to pro-angiogenic stimuli and hemodynamic force. The particular advantage of using HHT patient-derived hiPSC lines is that they can be engineered to allow inducible *ENG* knock down or degron-based ENG deletion. We expect that this, in combination with incorporation of pro-inflammatory triggers, such as pro-inflammatory macrophages, into the model will allow complete recapitulation of the phenotype in the future, such that these next-generation models can be implemented in screening for new therapeutic interventions and drug discovery using mechanism-based approaches with opportunities for validation using ECs from patient-derived hiPSCs.

EXPERIMENTAL PROCEDURES

HHT1 patient-derived hiPSC lines

Biopsies were taken with an informed consent at the St. Antonius Hospital (Nieuwegein, the Netherlands). The generation of the lines was approved by the Leiden University ethics committee under the P13.080 "Parapluprotocol: hiPSC." Patient samples, fibroblasts from skin biopsies, and erythroblasts isolated from peripheral blood were used for reprogramming. Reprogramming with episomal vectors was done as described, except that a newer generation of vectors without *TP53* shRNA were used (Okita et al., 2011). hiPSCs were routinely cultured on Matrigel (BD) in mTeSR1 and/or on Vitronectin XF in TeSR-E8 (all from Stem Cell Technologies) according to the manufacturer's protocol. Standard characterization of hiPSCs was performed as described previously (Bouma et al., 2017, 2020; Dambrot et al., 2013; Freund et al., 2010). Karyotype analysis of undifferentiated cells was performed using combined binary ratio labeling-fluorescence *in situ* hybridization (COBRA-FISH) (Szuhai and Tanke, 2006), and pluripotency of the hiPSC clones was confirmed by PluriTest, immunofluorescent staining for OCT3/4, SSEA-4, NANOG, and TRA-1-81, and spontaneous

(HHT$^{WT\#1}$, HHT$^{c.1678C>T\#1}$). From N = 5, n = 15; five independent experiments with three microfluidic channels per experiment (HHT$^{WT\#1}$, HHT$^{c.1678C>T\#2}$). Error bars are ± SD. Unpaired t test. $^{**}p < 0.005$.

(C) Representative spinning disk confocal images of vascular networks of HHT1-hiPSC-ECs differentiated from HHT1^{WT} and HHT1$^{c.1678C>T}$ hiPSC lines and HBVPs in 3D microfluidic chips with hiPSC-EC (magenta; CD31) and HBVPs (green; SM22). Scale bar: 100 μm.

(D) Left panels: surface rendering of images in (B) processed using IMARIS 9.5 software (Bitplane, Oxford Instruments). Scale bar: 100 μm. Right panels: magnification of the framed area in the left panel.

(E) Surface-rendering images processed using Imaris 9.5 software (Bitplane, Oxford Instruments) from spinning disk confocal images showing vascular networks formed by HHT1-hiPSC-ECs (gray; CD31) differentiated from HHT1^{WT} and HHT1$^{c.1678C>T}$ hiPSC lines in microfluidic chips. Color code for HBVPs showing green for objects touching the vessel, and color scale representing distance from the vessel. Scale bar: 30 μm.

(F) Quantification of average distance of surface-rendered SM22 cells to CD31 surface-rendered objects using IMARIS 9.5 software (Bitplane, Oxford Instruments). From N = 5, n = 7 (wild type [WT]) and n = 6 (mutant [MUT]); five independent experiments with one to three areas of each channel quantified. Error bars are ± SD. Unpaired t test. $^{**}p < 0.01$.

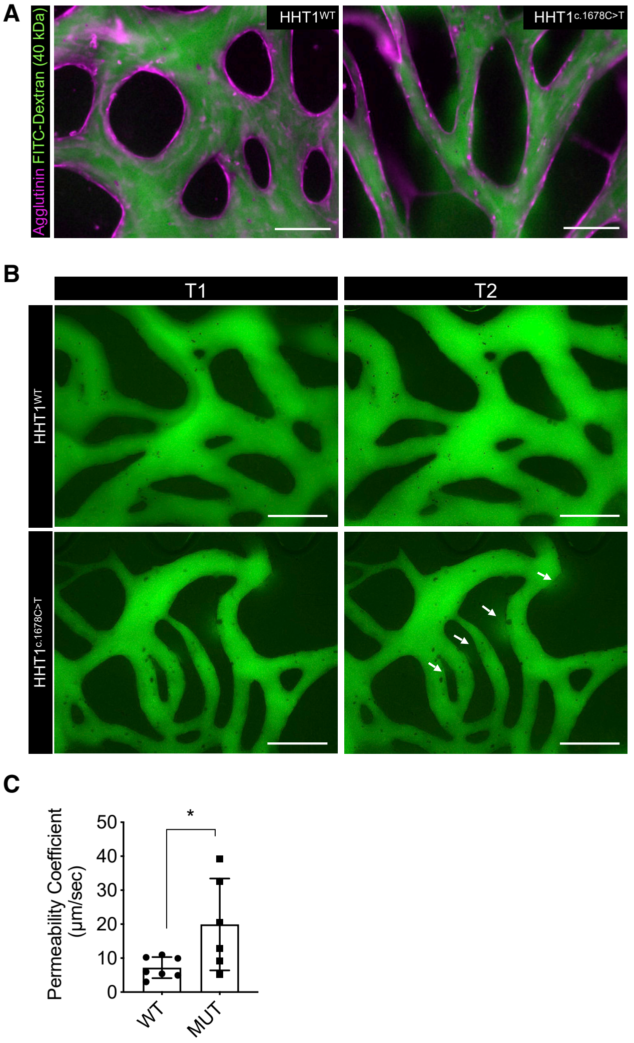

Figure 4. HHT1-hiPSC-ECs form leaky vessels

(A) Representative immunofluorescent images of vascular network formed by HHT1-hiPSC-ECs differentiated from HHT1^{WT} and HHT1$^{c.1678C>T}$ hiPSC lines and HBVPs in 3D microfluidic chips with hiPSC-EC (magenta; agglutinin) and dextran (40 kDa, green). 10x (EVOS) scale bar: 200 μm.

(B) Representative immunofluorescent images of vascular networks perfused with dextran (40 kDa, green) at two different timepoints (T1 = 0 s and T2 = 30 s). White arrows show dextran leakage in vascular network formed by HHT1$^{c.1678C>T}$ hiPSC-ECs.

(C) Quantification of permeability coefficient from N = 3, n = 7; three independent biological replicates with duplicate or triplicate microfluidic channels per experiment. Data are shown as ± SD. Unpaired t test. *$p < 0.05$.

differentiation toward three germ lineages. Sample identity has been confirmed by analysis with the DNA analysis software GeneMarker v.2.6.0 (SoftGenetics, State College, PA, USA) of fragments generated by the AmpFlSTR Profiler Plus PCR Amplification Kit (Applied Biosystems, Foster City, CA, USA) that have been run on a 3730 DNA Analyzer (Applied Biosystems). All tests were performed according to the instructions of the manufacturers.

Statistics

One-way ANOVA and non-parametric Student's t test for unpaired measurements were applied as appropriate to test for differences in means between the groups. Detailed statistics are indicated in each figure legend. Data are expressed and plotted as the mean ± SD. Statistical significance is indicated in each figure legend. Statistical analysis was performed with GraphPad Prism 9.0.2.

SUPPLEMENTAL INFORMATION

Supplemental information can be found online at https://doi.org/10.1016/j.stemcr.2022.05.022.

AUTHOR CONTRIBUTIONS

V.V.O., designed the research, established functional assays, performed experiments, and wrote the manuscript; D.M.N. and A.C., performed experiments in 3D vascular chips, did imaging, and performed quantification; X.C., performed EC differentiation; C.F., performed reprogramming experiments; F.v.d.H., conducted EC differentiation and isolation and FACS; F.L., assisted with quantification of microfluidic experiments; C.J.J.W., R.J.S., and H.-J.M., provided HHT patient samples; J.K.P.v.A., conducted genetic analysis; P.t.D. and F.L. helped analyze the data; C.L.M., designed the research and wrote the manuscript.

ACKNOWLEDGMENTS

The LUMC human iPSC Hotel generated and carried out initial characterization of hiPSC lines. The LUMC confocal imaging facility (Lennard Voortman, Annelies Boonzaier - van der Laan) are thanked for help with imaging; Karoly Szuhai and Danielle de Jong for help with COBRA-FISH; patients with HHT for their contribution to research; Lucas Hawikels and Gonzalo Sánchez-Duffhues for discussion; and Douwe Atsma for collecting patient samples. Illustrations were created using BioRender.com. This work was supported by the Netherlands Organization for Health Research and Development (ZonMw) PTO 446002501 in collaboration with CVON-PHAEDRA Impact; the Netherlands Organ-on-Chip Initiative is an NWO Gravitation project (024.003.001) funded by the Ministry of Education, Culture and Science of the government of the Netherlands; European Research Council (ERCAdG 323182 STEMCARDIOVASC); the Association Maladie de Rendu-Osler (AMRO) and the Kees Westermann Fund; and the European Union's Horizon 2020 research and innovation program under Marie Sklodowska Curie grant agreement no. 707404.

CONFLICT OF INTERESTS

The authors declare no competing interests.

Received: March 1, 2021
Revised: May 31, 2022
Accepted: May 31, 2022
Published: June 30, 2022

REFERENCES

Arthur, H.M., Ure, J., Smith, A.J.H., Renforth, G., Wilson, D.I., Torsney, E., Charlton, R., Parums, D.V., Jowett, T., Marchuk, D.A., et al. (2000). Endoglin, an ancillary TGFβ receptor, is required for extraembryonic angiogenesis and plays a key role in heart development. Dev. Biol. *217*, 42–53.

Begbie, M.E., Wallace, G.M.F., and Shovlin, C.L. (2003). Hereditary haemorrhagic telangiectasia (Osler-Weber-Rendu syndrome): a view from the 21st century. Postgrad. Med. *79*, 18–24.

Bouma, M.J., Iterson, M. van, Janssen, B., Mummery, C.L., Salvatori, D.C.F., and Freund, C. (2017). Differentiation-defective human induced pluripotent stem cells reveal strengths and limitations of the teratoma assay and in vitro pluripotency assays. Stem Cell Rep. *8*, 1340–1353.

Bouma, M.J., Orlova, V., Hil, F.E. van den, Mager, H.-J., Baas, F., Knijff, P. de, Mummery, C.L., Mikkers, H., and Freund, C. (2020). Generation and genetic repair of 2 iPSC clones from a patient bearing a heterozygous c.1120del18 mutation in the ACVRL1 gene leading to Hereditary Hemorrhagic Telangiectasia (HHT) type 2. Stem Cell Res. *46*, 101786.

Campisi, M., Shin, Y., Osaki, T., Hajal, C., Chiono, V., and Kamm, R.D. (2018). 3D self-organized microvascular model of the human blood-brain barrier with endothelial cells, pericytes and astrocytes. Biomaterials *180*, 117–129.

Carvalho, R.L.C., Jonker, L., Goumans, M.-J., Larsson, J., Bouwman, P., Karlsson, S., Dijke, P. ten, Arthur, H.M., and Mummery, C.L. (2004). Defective paracrine signalling by TGFβ in yolk sac vasculature of endoglin mutant mice: a paradigm for hereditary haemorrhagic telangiectasia. Development *131*, 6237–6247.

Chan, N.L.M., Bourdeau, A., Vera, S., Abdalla, S., Gross, M., Wong, J., Cymerman, U., Paterson, A.D., Mullen, B., and Letarte, M. (2004). Umbilical vein and placental vessels from newborns with hereditary haemorrhagic telangiectasia type 1 genotype are normal despite reduced expression of endoglin. Placenta *25*, 208–217.

Chen, M.B., Whisler, J.A., se, J.F., ouml, Y.,C., Shin, Y., and Kamm, R.D. (2017). On-chip human microvasculature assay for visualization and quantification of tumor cell extravasation dynamics. Nat. Protoc. *12*, 865–880.

Dambrot, C., Pas, S. van de, Zijl, L. van, Brändl, B., Wang, J.W., Schalij, M.J., Hoeben, R.C., Atsma, D.E., Mikkers, H.M., Mummery, C.L., et al. (2013). Polycistronic lentivirus induced pluripotent stem cells from skin biopsies after long term storage, blood outgrowth endothelial cells and cells from milk teeth. Differentiation; Research in Biological Diversity *85*, 101–109.

Dupuis-Girod, S., Ginon, I., Saurin, J.-C., Marion, D., Guillot, E., Decullier, E., Roux, A., Carette, M.-F., Gilbert-Dussardier, B., Hatron, P.-Y., et al. (2012). Bevacizumab in patients with hereditary hemorrhagic telangiectasia and severe hepatic vascular malformations and high cardiac output. JAMA *307*, 948–955.

El-Brolosy, M.A., and Stainier, D.Y.R. (2017). Genetic compensation: a phenomenon in search of mechanisms. PLoS Genet. *13*. e1006780–-17.

Fernandez-L, A., Sanz-Rodriguez, F., Zarrabeitia, R., Pérez-Molino, A., Hebbel, R.P., Nguyen, J., Bernabéu, C., and Botella, L.-M. (2005). Blood outgrowth endothelial cells from Hereditary Haemorrhagic Telangiectasia patients reveal abnormalities compatible with vascular lesions. Cardiovasc. Res. *68*, 235–248.

Franco, C.A., Jones, M.L., Bernabeu, M.O., Geudens, I., Mathivet, T., Rosa, A., Lopes, F.M., Lima, A.P., Ragab, A., Collins, R.T., et al. (2015). Dynamic endothelial cell rearrangements drive developmental vessel regression. PLoS Biol. *13*, e1002125.

Freund, C., Davis, R.P., Gkatzis, K., Oostwaard, D.W., and Mummery, C.L. (2010). The first reported generation of human induced pluripotent stem cells (iPS cells) and iPS cell-derived cardiomyocytes in The Netherlands. Neth. Heart J. *18*, 51–54.

Galaris, G., Thalgott, J.H., and Lebrin, F.P.G. (2019). Pericyte biology in disease. Adv. Exp. Med. Biol. *1147*, 215–246.

Goumans, M.-J., Liu, Z., and Dijke, P. ten (2009). TGF-β signaling in vascular biology and dysfunction. Cell Res. *19*, 116–127.

Govani, F.S., and Shovlin, C.L. (2009). Hereditary haemorrhagic telangiectasia: a clinical and scientific review. Eur. J. Hum. Genet. *17*, 860–871.

Halaidych, O.V., Freund, C., Hil, F. van den, Salvatori, D.C.F., Riminucci, M., Mummery, C.L., and Orlova, V.V. (2018). Inflammatory responses and barrier function of endothelial cells derived from human induced pluripotent stem cells. Stem Cell Rep. *10*, 1642–1656.

Kochhan, E., Lenard, A., Ellertsdottir, E., Herwig, L., Affolter, M., Belting, H.-G., and Siekmann, A.F. (2013). Blood flow changes coincide with cellular rearrangements during blood vessel pruning in zebrafish embryos. PLoS One *8*, e75060.

Kroon, S., Snijder, R.J., Hosman, A.E., Vorselaars, V.M.M., Disch, F.J.M., Post, M.C., and Mager, J.J. (2020). Oral itraconazole for epistaxis in hereditary hemorrhagic telangiectasia: a proof of concept study. Angiogenesis *24*, 379–386.

Laake, L.W. van, Driesche, S. van den, Post, S., Feijen, A., Jansen, M.A., Driessens, M.H., Mager, J.J., Snijder, R.J., Westermann, C.J.J., Doevendans, P.A., et al. (2006). Endoglin has a crucial role in blood cell-mediated vascular repair. Circulation *114*, 2288–2297.

Lebrin, F., Goumans, M.-J., Jonker, L., Carvalho, R.L.C., Valdimarsdottir, G., Thorikay, M., Mummery, C., Arthur, H.M., and Dijke, P. ten (2004). Endoglin promotes endothelial cell proliferation and TGF-β/ALK1 signal transduction. EMBO J. *23*, 4018–4028.

Lebrin, F., Srun, S., Raymond, K., Martin, S., Brink, S. van den, Freitas, C., Bréant, C., Mathivet, T., Larrivée, B., Thomas, J.-L., et al. (2010). Thalidomide stimulates vessel maturation and reduces epistaxis in individuals with hereditary hemorrhagic telangiectasia. Nat. Med. *16*, 420–428.

Letteboer, T.G.W., Zewald, R.A., Kamping, E.J., Haas, G. de, Mager, J.J., Snijder, R.J., Lindhout, D., Hennekam, F.A.M., Westermann, C.J.J., and Amstel, J.K.P. van (2005). Hereditary hemorrhagic telangiectasia: ENG and ALK-1 mutations in Dutch patients. Hum. Genet. *116*, 8–16.

Mahmoud, M., Allinson, K.R., Zhai, Z., Oakenfull, R., Ghandi, P., Adams, R.H., Fruttiger, M., and Arthur, H.M. (2010). Pathogenesis of arteriovenous malformations in the absence of endoglin. Circ. Res. *106*, 1425–1433.

Okita, K., Matsumura, Y., Sato, Y., Okada, A., Morizane, A., Okamoto, S., Hong, H., Nakagawa, M., Tanabe, K., Tezuka, K., et al. (2011). A more efficient method to generate integration-free human iPS cells. Nat. Methods *8*, 409–412.

Orlova, V.V., Drabsch, Y., Freund, C., Petrus-Reurer, S., Hil, F.E. van den, Muenthaisong, S., Dijke, P. ten, and Mummery, C.L. (2014a). Functionality of endothelial cells and pericytes from human pluripotent stem cells demonstrated in cultured vascular plexus and zebrafish xenografts. Arterioscler. Thromb. Vasc. Biol. *34*, 177–186.

Orlova, V.V., Hil, F.E. van den, Petrus-Reurer, S., Drabsch, Y., Dijke, P. ten, and Mummery, C.L. (2014b). Generation, expansion and functional analysis of endothelial cells and pericytes derived from human pluripotent stem cells. Nat. Protoc. *9*, 1514–1531.

Shin, Y., Han, S., Jeon, J.S., Yamamoto, K., Zervantonakis, I.K., Sudo, R., Kamm, R.D., and Chung, S. (2012). Microfluidic assay for simultaneous culture of multiple cell types on surfaces or within hydrogels. Nat. Protoc. *7*, 1247–1259.

Shovlin, C.L. (2010). Hereditary haemorrhagic telangiectasia: pathophysiology, diagnosis and treatment. Blood Rev. *24*, 203–219.

Snodgrass, R.O., Chico, T.J.A., and Arthur, H.M. (2021). Hereditary haemorrhagic telangiectasia, an inherited vascular disorder in need of improved evidence-based pharmaceutical interventions. Genes-Basel *12*, 174.

Sugden, W.W., and Siekmann, A.F. (2018). Endothelial cell biology of Endoglin in hereditary hemorrhagic telangiectasia. Curr. Opin. Hematol. *25*, 1–8.

Sugden, W.W., Meissner, R., Aegerter-Wilmsen, T., Tsaryk, R., Leonard, E.V., Bussmann, J., Hamm, M.J., Herzog, W., Jin, Y., Jakobsson, L., et al. (2017). Endoglin controls blood vessel diameter through endothelial cell shape changes in response to haemodynamic cues. Nat. Cell Biol. *19*, 653–665.

Szuhai, K., and Tanke, H.J. (2006). COBRA: combined binary ratio labeling of nucleic-acid probes for multi-color fluorescence in situ hybridization karyotyping. Nat. Protoc. *1*, 264–275.

Tual-Chalot, S., Oh, S.P., and Arthur, H.M. (2015). Mouse models of hereditary hemorrhagic telangiectasia: recent advances and future challenges. Front. Genet. *6*, 25.

Vila Cuenca, M., Cochrane, A., Hil, F.E. van den, Vries, A.A.F. de, Oberstein, S.A.J.L., Mummery, C.L., and Orlova, V.V. (2021). Engineered 3D vessel-on-chip using hiPSC-derived endothelial- and vascular smooth muscle cells. Stem Cell Rep. *16*, 2159–2168.

Yamamoto, H., Ehling, M., Kato, K., Kanai, K., Lessen, M. van, Frye, M., Zeuschner, D., Nakayama, M., Vestweber, D., and Adams, R.H. (2015). Integrin β1 controls VE-cadherin localization and blood vessel stability. Nat. Commun. *6*, 6429.

Stem Cell Reports, Volume *17*

Supplemental Information

Vascular defects associated with hereditary hemorrhagic telangiecta-sia revealed in patient-derived isogenic iPSCs in 3D vessels on chip

Valeria V. Orlova, Dennis M. Nahon, Amy Cochrane, Xu Cao, Christian Freund, Francijna van den Hil, Cornelius J.J. Westermann, Repke J. Snijder, Johannes Kristian Ploos van Amstel, Peter ten Dijke, Franck Lebrin, Hans-Jurgen Mager, and Christine L. Mummery

Inventory of Supplemental Information

Supplemental Figures and Legends:
Figure S1. Related to Figure 1. Characterization of HHT1 patient-derived hiPSCs.
Figure S2. Related to Figure 1. Characterization of HHT1-hiPSC-ECs.
Figure S3. Related to Figure 2. HHT1-hiPSC-ECs show defective vascular organization in VoC.
Figure S4. Related to Figure 3. HHT1-hiPSC-ECs show defective EC-pericyte cell interaction in VoC.

Supplemental Tables:

Supplemental Table 1. List of FACS antibodies.
Supplemental Table 2. List of antibodies for IF.

Supplemental Videos:

Supplemental Video 1. Perfusion of fluorescent beads in 3D vessels formed by HHT1^{WT}-hiPSC-ECs and HHT1$^{c.1678C>T}$-hiPSC-ECs. Related to Figure 2.

Supplemental Video 2. Perfusion of FITC-Dextran (40 kDa) in 3D vessels formed by HHT1^{WT}-hiPSC-ECs and HHT1$^{c.1678C>T}$-hiPSC-ECs. Related to Figure 4.

Supplemental Experimental Procedures

Supplemental References

SUPPLEMENTAL FIGURE 1.

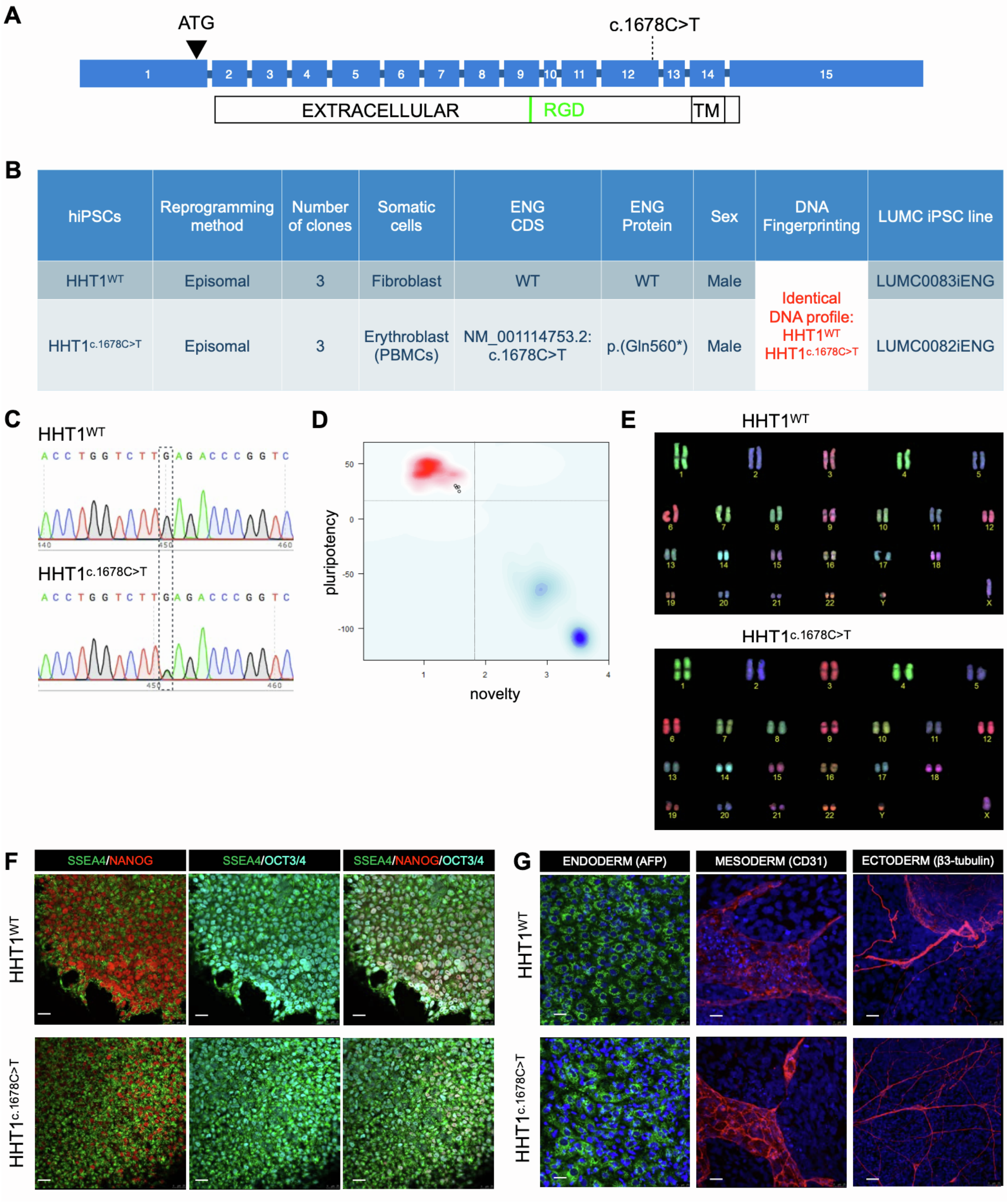

hiPSCs	Reprogramming method	Number of clones	Somatic cells	ENG CDS	ENG Protein	Sex	DNA Fingerprinting	LUMC iPSC line
HHT1WT	Episomal	3	Fibroblast	WT	WT	Male	Identical DNA profile: HHT1WT HHT1c.1678C>T	LUMC0083iENG
HHT1c.1678C>T	Episomal	3	Erythroblast (PBMCs)	NM_001114753.2: c.1678C>T	p.(Gln560*)	Male		LUMC0082iENG

Figure S1. Related to Figure 1. Characterization of HHT1 patient-derived hiPSCs. (A) Schematic overview of *ENG* genomic map, protein map and location of the mutation. (B) Overview of HHT-hiPSC lines. (C) Sanger sequencing of the genomic DNA from HHT1 patient-derived hiPSC lines to confirm the mutation. (D) Bioinformatic assessment of pluripotency of HHT1 patient-derived hiPSCs (PluriTest): two clones per hiPSC line (HHT1^{WT} and HHT1$^{c.1678C>T}$ hiPSCs) were analyzed. (E) COBRA-FISH analysis of karyotype of HHT1 patient-derived hiPSCs. (F) Immunofluorescent images of expression of pluripotency markers: OCT3/4, SSEA4 and Nanog. Scale bar 25 μm. (G) Immunofluorescent images of the spontaneous differentiation demonstrating derivatives of all three germ layers: βIII-tubulin for neuroectoderm, CD31 for mesoderm, AFP for endoderm. Scale bar 25μm.

SUPPLEMENTAL FIGURE 2.

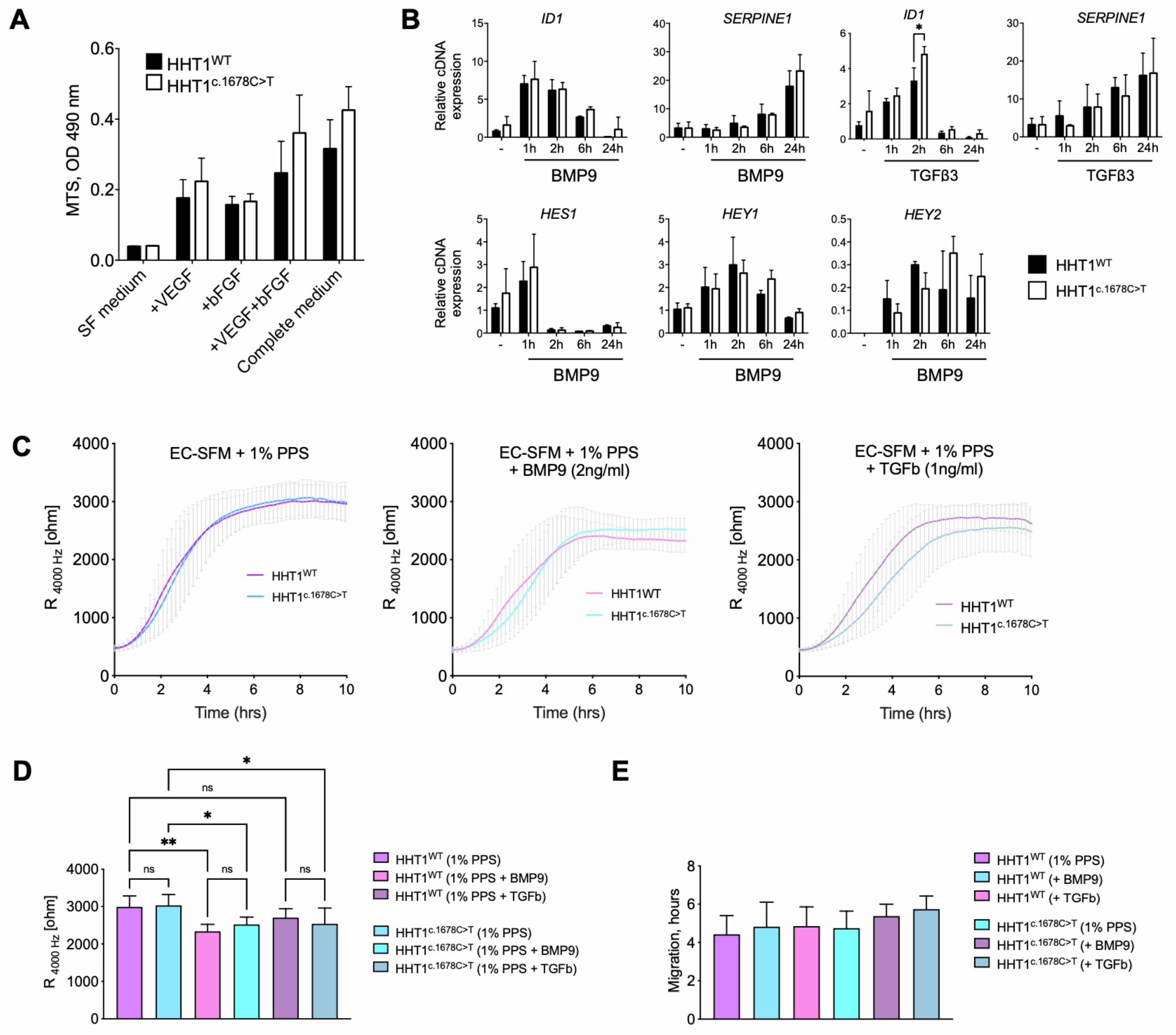

Figure S2. Related to Figure 1. Characterization of HHT1-hiPSC-ECs. (A) Assessment of EC proliferation in serum-free medium (SF medium), SF medium + VEGF (+VEGF), SF medium + bFGF (+FGF), SF medium VEGF + bFGF (+VEGF+FGF) or complete EC growth medium (Complete medium) at 72h after medium change. The analysis was performed on ECs differentiated from three independent clones of HHT1 patient-derived isogenic hiPSCs. Error bars are ±SD. (B) Gene expression analysis of expression of *ID1, SERPINE1, HES1, HEY1 and HEY2* (upon BMP9 1ng/ml stimulation) and *ID1, SERPINE1* (upon TGFβ 1ng/ml stimulation). ECs differentiated from two independent hiPSC clones were analyzed. Data are shown ±SD from three independent experiments. One-way ANOVA. *p<0.05 (C) Absolute resistance of the EC monolayer at 4000 Hz in growth factor-free medium supplemented with 1% PPS (1% PPS), or 1% PPS supplemented with BMP9 (2 ng/ml)(+ BMP9) or 1% PPS supplemented with TGFβ (1 ng/ml)(+ TGFβ) is shown. ECs differentiated from two independent hiPSC clones were analysed. Error bars are ±SD of six independent biological experiments. (D) Quantification of absolute resistance values at 4000 Hz from C. Error bars are shown as ±SD of five independent biological experiments. (E) Quantification of migration rates. Error bars are shown as ±SD of six independent biological experiments. One-way ANOVA. **p<0.01, *p<0.05.

SUPPLEMENTAL FIGURE 3.

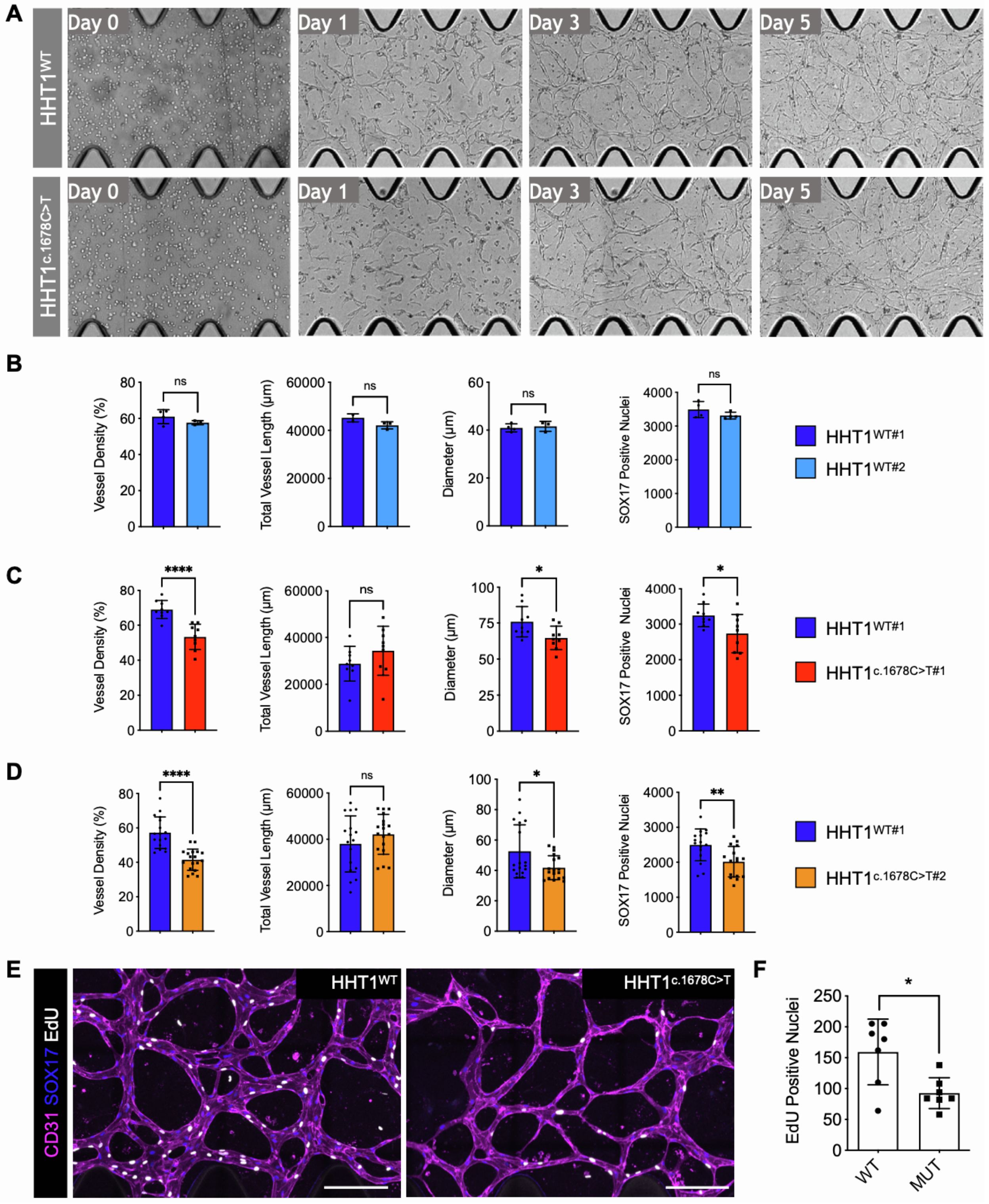

Figure S3. Related to Figure 2. HHT1-hiPSC-ECs show defective vascular organization in VoC. (A) Representative images of microfluidic chips seeded with ECs from HHT1 patient-derived isogenic hiPSCs. Images were taken every 24h from day 0 (after seeding) till day 5. (B) Quantification of HHT1-hiPSC-EC vascular network showing vessel density (%), total vessel length (μm), diameter (μm) and number of HHT1-hiPSC-ECs (SOX17+ nuclei). Data are shown as ±SD, Unpaired t-test. ns = not significant. From N=1, n=3; one independent biological experiment with three microfluidic channels. (C,D) Quantification of HHT1-hiPSC-EC vascular network showing vessel density (%), total vessel length (μm), diameter (μm) and number of HHT1-hiPSC-ECs (SOX17+ nuclei). Data are shown as ±SD, Unpaired t-test. **** $p<0.001$, *$p < 0.05$, ns = not significant. From N=3, n=9; three independent biological experimnts with three microfluidic channels per experiment (C). From N=5, n=15; five independent biological experiments (with three microfluidic channels per experiment (D). (E) Representative images showing proliferative (EdU positive) (white) HHT1-hiPSC-ECs differentiated from $HHT1^{WT}$ and $HHT1^{c.1678C>T}$ hiPSC lines and HBVPs in 3D microfluidic chips. Scale bar represents 200 μm. (F) Quantification of proliferative (EdU positive) HHT1-hiPSC-ECs in 3D vascular network in the microfluidic chip. Error bars are ±SD of four microfluidic cultures (one independent biological experiment). Unpaired t-test. * $p<0.05$.

SUPPLEMENTAL FIGURE 4.

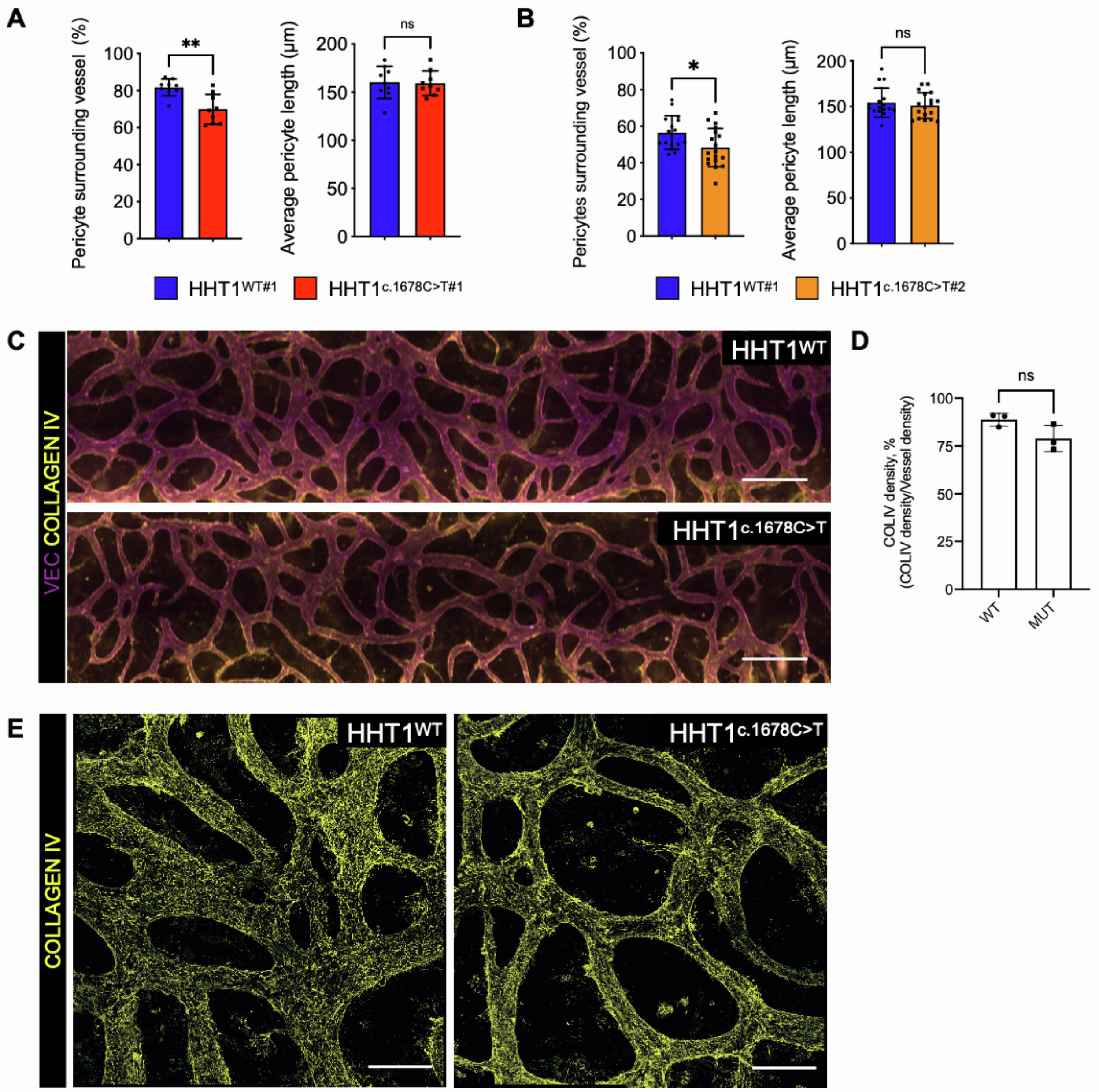

Figure S4. Related to Figure 3. HHT1-hiPSC-ECs show defective EC-pericyte cell interaction in VoC. (A, B) Quantification of % pericytes surrounding vessel and average length of pericytes using CellProfiler. From N=3, n=9; three independent biological experiments with mean from triplicate microfluidic channels per experiment (A). From N=5, n=15; five independent biological experiments with mean from triplicate microfluidic channels per experiment (B). Error bars are ±SD, Unpaired t-test. * $p<0.05$. (C) Representative images of vascular networks formed by HHT1-hiPSC-ECs differentiated from HHT1^{WT} and HHT1$^{c.1678C>T}$ hiPSC lines in microfluidic chips. ECs are stained with anti-VEC (magenta) and anti-COLIV (yellow). Scale bar represents 500 µm. (D) Quantification of collagen IV density (%). Data are shown as ±SD from N=1, n = 3; one independent biological experiment, three microfluidic channels. Unpaired t-test. ns = not significant. (E) Representative spinning disk confocal images showing HHT1-hiPSC-EC vascular network stained for ECM (yellow; COLLAGEN IV). Scale bar 30 µm.

Supplemental Tables

Supplemental Table 1. List of FACS antibodies

Antibody	Fluorochrome	Source	Dilution	Catalog #
VE-Cadherin	A488	eBiosciences	1:50	53-1449-42
CD31	APC	eBiosciences	1:100	17-0319-42
KDR	PE	R&D systems	1:20	FAB357P
ENG	VioBlue	Miltenyi Biotec	1:20	130-099-666
ENG	PE-Vio-770	Miltenyi Biotec	1:20	130-099-889

Supplemental Table 2. List of antibodies for IF

Antibody	Species	Source	Dilution	Catalog #
SSEA-4	Mouse	Biolegend	1:30	330402
Nanog	Mouse	Santa Cruz	1:150	sc-293121
OCT3/4	Mouse	Santa Cruz	1:100	sc-5279
AFP	Rabbit	Quartett	1:25	2011200530
CD31	Mouse	Dako	1:200	M0823
βIII-tubulin	Mouse	Covance	1:4000	MMS-435P
VEC	Rabbit	CellSignaling	1:200	2158S
vWF	Rabbit	Dako	1:200	A0082
SOX17	Goat	R&D systems	1:200	AF1924
SM22	Rabbit	Abcam	1:200	AB14106
Collagen IV	Goat	Sigma	1:200	AB769
Laminin	Rabbit	Merck	1:100	AB19012
Alexa Fluor 488	Mouse IgG	Invitrogen	1:500	A11001
Alexa Fluor 568	Mouse IgM	Invitrogen	1:200	A21043
Alexa Fluor 488	Mouse IgG3	Invitrogen	1:250	A21151
Alexa Fluor 568	Mouse IgG1	Invitrogen	1:250	A21124
Alexa Fluor 647	Mouse IgG2b	Invitrogen	1:250	A21242
Alexa Fluor 568	Mouse IgG	Invitrogen	1:500	A11031
Alexa Fluor 488	Rabbit IgG	Invitrogen	1:500	A21206

Supplemental Experimental Procedures

Sanger sequencing

ENG mutations were confirmed by Sanger sequencing. DNA was extracted using the Gentra Puregene Cell Kit (QIAGEN) according to the manufacturer's protocol. PCR was performed to amplify *ENG* exon 12 (FW: CCAGAGTCAGGAGGGAGACA; RV: GCGTCCAGGATAGATTGCCT, Product size: 974 bp). The PCR products were purified using the QIAquick PCR Purification Kit and were send for Sanger sequencing (BaseClear).

Differentiation of hiPSC-ECs

hiPSC differentiation towards endothelial cells and CD31 magnetic bead isolation were performed as described previously (Halaidych et al., 2018; Orlova et al., 2014a, 2014b). Briefly, hiPSCs were passaged in normal culture conditions one day before inducing differentiation. Mesoderm differentiation was induced by changing the media to B(P)EL with a high concentration CHIR99021 (8 μM). At day 3, 6 and 9 of

differentiation the cells were refreshed with B(P)EL with VEGF (50 ng/ml) and SB43152 (10 µM, Tocris). ECs were purified with CD31 coupled magnetic beads at day 10 (Life Technologies) and the culture was further scaled up on 0,1% gelatin coated tissue culture flasks in human endothelial serum free media (EC-SFM)(Life Technologies) with additional VEGF (30 ng/ml), bFGF (20 ng/ml, R&D) and 1% platelet poor serum (PPS)(Hycultec). Functional assays were performed on cells between passages 2-3.

FACS analysis of hiPSC-ECs
FACS analysis was performed as described previously (Halaidych et al., 2018; Orlova et al., 2014a, 2014b). Purified hiPSC-ECs were dissociated with TrypLE Select (Life Technologies). Combinations of the following antibodies were used in flow cytometry experiments (see supplemental table 1): VE-Cadherin-A488 (1:50, eBiosciences), CD31-APC (1:100, eBiosciences), KDR-PE (1:20, R&D Systems), ENG-VioBlue or PE-Vio-770 (1:20, Milteny Biotec). Samples were analysed either on LSRII (BD) with the following instrument setup: Blue/488 FITC, A488: 505LP-530/30, PerCP-Cy5.5: 630LP-670/14; Yellow/561 PE: 570LP-582/15, APC: 635LP-660/20; or on MACSQuant VYB (Miltenyi) with the following instrument setup: Blue/488 FITC, A488: 525/50; Yellow/561 PE: 586/15, APC: 661/20, APC-Cy7: 750LP.

Immunofluorescent staining of hiPSC-ECs
Immunofluorescent staining was performed as described previously (Halaidych et al., 2018; Orlova et al., 2014a, 2014b). Briefly, ECs were seeded on FN-coated 96-well black imaging plates (Corning) at the seeding density ~10,000cells/well in complete EC growth culture medium (EC-SFM supplemented with VEGF 30 ng/ml, bFGF 20 ng/ml and 1% PPS). 48h post-seeding ECs were fixed with 4% paraformaldehyde (PFA, Sigma), permeabilized with the 0.1%TX-100 and stained with anti-CD31 (1:200, Dako) and anti-vWF (1:200, Dako) or VEC (CellSignaling)(supplemental table 2). High magnification images were acquired with the WLL1 confocal microscope (Leica), using 40x DRY objective.

Endothelial cell proliferation (MTS assay)
hiPSC-ECs were seeded into on FN-coated 96-well plates at the seeding density 2,000 cells/well in EC-SFM for 12 h and subsequently refreshed with EC-SFM containing various stimuli. After 4 days the MTS assay (CellTiter, Promega) was used to determine the relative number of ECs.

Assessment of hiPSC-ECs functionality in an *in vitro* vasculogenesis assay
The co-culture experiments with hiPSC-ECs or primary ECs and stromal cells were performed as described previously (Halaidych et al., 2018; Orlova et al., 2014a, 2014b). The co-cultures were stopped at day 10 and post-fixed and stained with anti-CD31 (1:200, Dako) and anti-SOX17 (1:200, R&D), and anti-SM22 (1:200, Abcam) antibodies (supplemental table 2). The co-cultures were imaged with the EVOS FL AUTO2 Imaging system (ThermoFischer Scientific) with the 10X Objective for quantifications with autofocus on CD31, and auto stitching 4X4 focus planes or 20X Objective for CD31 and SOX17 images. The co-cultures were quantified using publicly available software AngioTool (Zudaire et al., 2011).

Endothelial barrier function analysis
Endothelial barrier function was measured using impedance-based cell monitoring with an electric cell-substrate impedance sensing system (ECIS Zθ, Applied Biophysics), as described previously (Halaidych et al., 2018). hiPSC-ECs were seeded on FN-coated ECIS arrays each containing 8 wells with 10 gold electrodes per well (8W10E PET, Applied Biophysics). The cell seeding density was estimated ~50,000cells/cm^2. For barrier function and migration studies the cells were seeded for at least 24h in complete EC growth medium followed by 6h serum starvation step in EC-SFM. For the assessment of cell migration after serum starvation, the medium was changed to complete EC growth medium or EC-SFM supplemented with 1% PPS, BMP9 (2ng/ml) and TGFβ3 (1ng/ml), and electric wound (10 sec pulse of 5V at 60 kHz) was applied to the cells 1h after medium change. Recovery of the barrier was monitored in real time over 6-12h. Multiple frequency/time (MFT) mode was used for the real-time assessment of the barrier and monolayer confluence.

Generation of perfused vascular networks in microfluidic chips
Vascular networks were generated as described previously (Chen et al., 2017) with some adjustments that were developed during optimization of the protocol with hiPSC-ECs and primary human brain vascular pericytes (HBVPs)(ScienceCell), and microfluidic chips with one gel channel and two media channels (AIM

Biotech). Cells were resuspended in EGM-2 supplemented with thrombin (4 U/ml) at $10x10^6$ cells/ml for hiPSC-ECs and $0.5x10^6$ cells/ml for HBVPs or $2x10^6$ cells/ml for HBVPs (note: higher HBVP numbers promote vessel formation, although earlier experiments were conducted with a lower number of HBVPs with comparable results). The cell suspension was mixed with an equal volume of fibrinogen solution (10 mg/ml; final concentration 5 mg/ml) and injected into the gel channel of the microfluidic chip; this t was left for 15 min at room temperature (RT) to allow fibrin gel to form. EGM-2 supplemented with VEGF (50 ng/ml) was added to each of the flanking media channels. Interstitial flow through the gel was achieved by adding a larger volume of medium to one of the media inlets, generating a pressure gradient. The microfluidic chips were refreshed every 24 hours with EGM-2 supplemented with VEGF (50 ng/ml) and γ-secretase inhibitor N-[N-(3,5-difluorophenacetyl)-l-alanyl]-s-phenylglycinet-butyl ester (DAPT, 10 μM) (DAPT supplementation was performed on day 1 for 24 hours). For immunofluorescent staining, 3D cultures were fixed with 4% paraformaldehyde (PFA; Sigma) for 20 min at RT, permeabilized with 0.5% TX-100 for 15 min at RT and blocked with 3% bovine serum albumin (BSA) in PBS for 3 hours at RT. Samples were stained by anti-CD31 (1:200, Dako), anti-SOX17(1:200, R&D), and anti-SM22 (1:200, Abcam). Details of antibodies are given in supplemental table 2. Primary antibodies were prepared in 2% BSA and incubated overnight at 4°C and secondary antibodies were prepared in 2% BSA and incubated for 2 hours at RT. Fluorescence images for quantification were acquired using EVOS AUTO2 using 10x magnification objective and high magnification images were acquired with a DragonFly spinning disk (Andor) microscope using 40x magnification objective post-processing, performed and processed using Imaris 9.5 software (Bitplane, Oxford Instruments).

EdU assay for EC proliferation in 3D microfluidic chips

Proliferation was measured using an EdU Click-iT kit Alexa-488 (ThermoFisher Scientific #C10337) according to manufacturer's protocol. Briefly, on day 6 of culture, microfluidic chips were refreshed with EGM-2 (VEGF 50 ng/ml and DAPT 10 μM) additionally supplemented with EdU (1:1000) for 8 hours. Cells were fixed with 4% PFA for 30 minutes, permeabilised with 0.5% TX-100 for 15 minutes at RT. Freshly prepared Click-iT reaction cocktail was added for 30 minutes at RT. Microfluidic chips were washed twice with 3% BSA-PBS and blocked in 3% BSA-PBS for 1-2 hours at RT, followed by co-staining with primary and secondary antibodies.

Quantification of 3D vessels in microfluidic chips

Microfluidic chips were imaged with the EVOS FL AUTO2 or M7000 Imaging system (ThermoFischer Scientific) using the 10X Objective with autofocus either on the cells in phase-contrast mode (for time series experiments) or on CD31 for fixed samples, and automatic image stitching to cover the entire gel channel for quantification of microvascular networks. Parameters were quantified using pipelines developed on the free open source CellProfiler software (https://cellprofiler.org/) (Carpenter et al., 2006). Briefly, for EC nuclei number, pre-processing steps were used to enhance image features and filter non-specific object identification. A Gaussian filter was applied to mural cell images before object identification was used to measure object morphology and interaction with hiPSC-EC network. Filter steps were applied to images of vascular network to reduce non-specific segmentation from cell junction staining and a minimum cross-entropy thresholding method was used to produce a binarized image. The binarized images from the CellProfiler output were then analysed using freely available ImageJ software with the plugin (https://imagej.nih.gov/ij/, https://imagej.net/DiameterJ) (Hotaling et al., 2015). For ECM quantification, binarized vessel images were used as masks to ensure Collagen IV staining intensity was quantified only at the vessel regions excluding any background noise. The number of EdU and SOX17 positive nuclei were quantified with a custom-made pipeline in CellProfiler (Carpenter et al., 2006). Additional quantification of the distance between SM22- and CD31 positive cells was obtained using Imaris 9.5 software (Bitplane, Oxford Instruments).

Perfusion assessment in vessels in microfluidic chips

For perfusion assessment, the chip was placed on day 6 in the EVOS AUTO2 with on stage incubator for time-lapse image acquisition. First, basal fluorescence activity was captured before the addition of fluorescent tracers. Next, 70 μl of 40KDa FITC-Dextran (1:1000, Sigma) or 405-beads (1:10, Fluoro-Max Dyed Blue Aqueous Fluorescent Particles, B0200, ThermoFisher Scientific) in EGM-2 was added to one medium port and 50 μl of EGM-2 to all other media ports to induce interstitial gravity flow. Then, images were captured simultaneously at 20 fps using a 10x magnification objective for 30 seconds. For

quantification of permeability coefficient, images acquired at T1 (time 0 seconds) and T2 (30 seconds of perfusion) were quantified using pipelines developed with CellProfiler (Carpenter et al., 2006). Briefly, a mask for vessel area inside image acquired was created to ensure intensity was measured in area outside the vessel wall only. Calculation for permeability coefficient was based on the previously established method (Campisi et al., 2018).

Supplemental References

Campisi, M., Shin, Y., Osaki, T., Hajal, C., Chiono, V., and Kamm, R.D. (2018). 3D self-organized microvascular model of the human blood-brain barrier with endothelial cells, pericytes and astrocytes. Biomaterials *180*, 117–129.

Carpenter, A.E., Jones, T.R., Lamprecht, M.R., Clarke, C., Kang, I.H., Friman, O., Guertin, D.A., Chang, J.H., Lindquist, R.A., Moffat, J., et al. (2006). CellProfiler: image analysis software for identifying and quantifying cell phenotypes. Genome Biology *7*, R100.

Chen, M.B., Whisler, J.A., se, J.F. ouml, Yu, C., Shin, Y., and Kamm, R.D. (2017). On-chip human microvasculature assay for visualization and quantification of tumor cell extravasation dynamics. Nature Protocols *12*, 865–880.

Halaidych, O.V., Freund, C., Hil, F. van den, Salvatori, D.C.F., Riminucci, M., Mummery, C.L., and Orlova, V.V. (2018). Inflammatory Responses and Barrier Function of Endothelial Cells Derived from Human Induced Pluripotent Stem Cells. Stem Cell Reports *10*, 1642–1656.

Hotaling, N.A., Bharti, K., Kriel, H., and Simon, C.G. (2015). DiameterJ: A validated open source nanofiber diameter measurement tool. Biomaterials *61*, 327–338.

Orlova, V.V., Drabsch, Y., Freund, C., Petrus-Reurer, S., Hil, F.E. van den, Muenthaisong, S., Dijke, P. ten, and Mummery, C.L. (2014a). Functionality of endothelial cells and pericytes from human pluripotent stem cells demonstrated in cultured vascular plexus and zebrafish xenografts. Arteriosclerosis, Thrombosis, and Vascular Biology *34*, 177–186.

Orlova, V.V., Hil, F.E. van den, Petrus-Reurer, S., Drabsch, Y., Dijke, P. ten, and Mummery, C.L. (2014b). Generation, expansion and functional analysis of endothelial cells and pericytes derived from human pluripotent stem cells. Nature Protocols *9*, 1514–1531.

Zudaire, E., Gambardella, L., Kurcz, C., and Vermeren, S. (2011). A Computational Tool for Quantitative Analysis of Vascular Networks. PLoS ONE *6*, e27385-12.
